# Addressing the challenges of infectious bone defects: a review of recent advances in bifunctional biomaterials

**DOI:** 10.1186/s12951-025-03295-0

**Published:** 2025-03-29

**Authors:** Huaiyuan Zhang, Wenyu Qiao, Yu Liu, Xizhou Yao, Yonghua Zhai, Longhai Du

**Affiliations:** 1 Department of Orthopedics, Jinshan Hospital, Fudan University, Shanghai, 201508 China; 2 Department of Cardiovascular Medicine, Department of Hypertension, Ruijin Hospital and State Key Laboratory of Medical Genomics, Shanghai Key Laboratory of Hypertension, Shanghai Institute of Hypertension, Shanghai Jiao Tong University School of Medicine, 197 Ruijin 2nd Road, Shanghai, 200025 China; 3 Department of General Surgery, Jinshan Hospital, Fudan University, Shanghai, 201508 China; 4 Research Center for Clinical Medicine, Jinshan Hospital Affiliated to Fudan University, Shanghai, 201508 China

**Keywords:** Infectious bone defects, Bifunctional materials, Bone tissue engineering, Antibacterial properties, Osteogenesis

## Abstract

Infectious bone defects present a substantial clinical challenge due to the complex interplay between infection control and bone regeneration. These defects often result from trauma, autoimmune diseases, infections, or tumors, requiring a nuanced approach that simultaneously addresses infection and promotes tissue repair. Recent advances in tissue engineering and materials science, particularly in nanomaterials and nano-drug formulations, have led to the development of bifunctional biomaterials with combined osteogenic and antibacterial properties. These materials offer an alternative to traditional bone grafts, minimizing complications such as multiple surgeries, high antibiotic dosages, and lengthy recovery periods. This review examines the repair mechanisms in the infectious microenvironment and highlights various bifunctional biomaterials that foster both anti—infective and osteogenic processes. Emerging design strategies are also discussed to provide a forward-looking perspective on treating infectious bone defects with clinically significant outcomes.

**Graphical Abstract:**

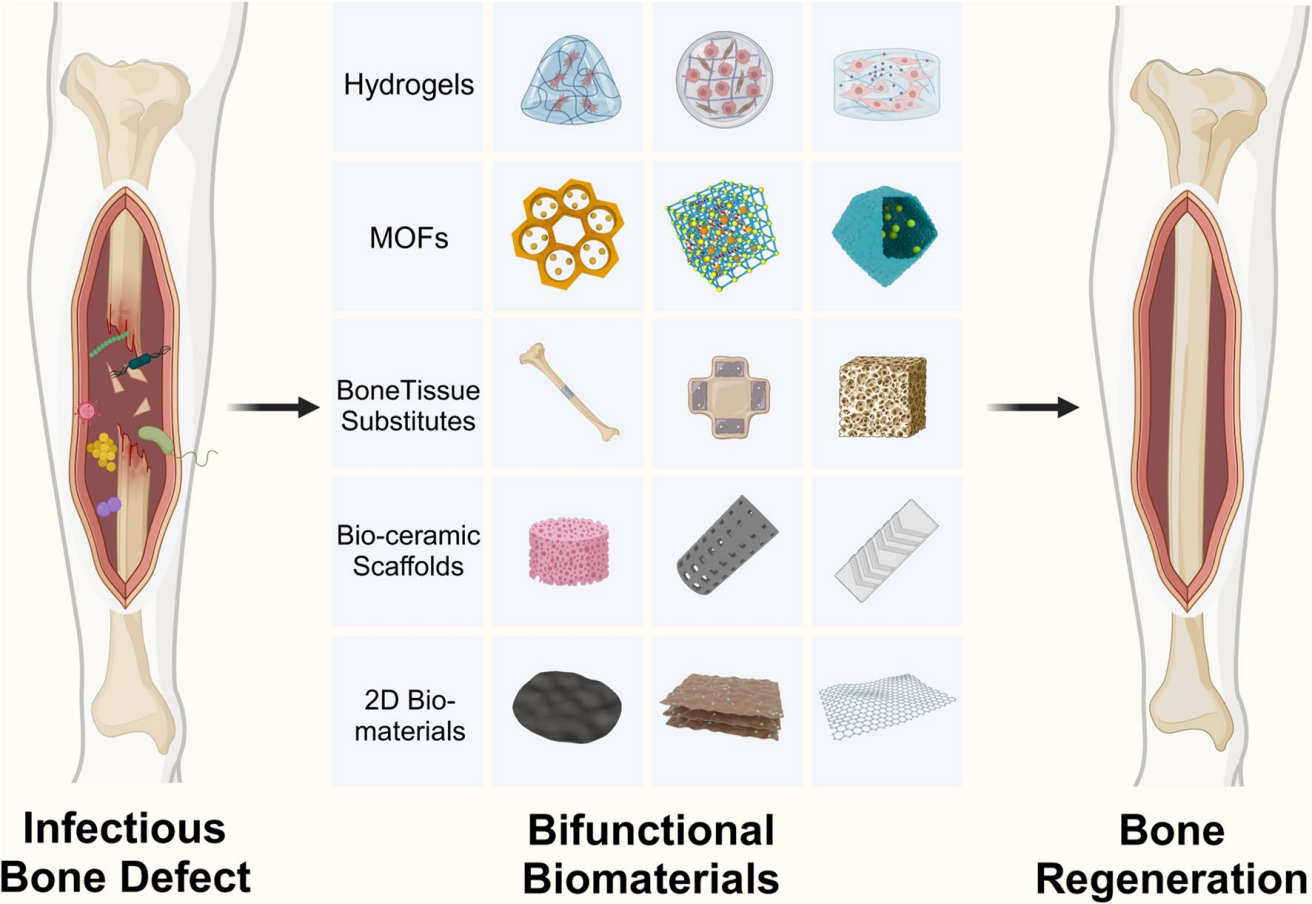

## Highlights

- Discussed the mechanisms of pathogenesis in infectious bone defects.
- Bifunctional biomaterials coordinate anti-infection and osteogenesis.
- Focused on the design of several bifunctional biomaterials.
- Explored the challenges and future prospects in the design and application of bifunctional biomaterials.

## Introduction

Bone tissue, a vital component of the human body, supports movement, protects organs, and serves as a primary site for hematopoiesis within bone marrow [1]. Structurally, bone is a dynamic composite material composed of organic and inorganic elements, containing living cells, extracellular matrix (ECM), blood vessels, and immune components, which together provide its remarkable regenerative capacity [2].

Bone defects caused by acute trauma (such as fractures), inflammatory diseases (including autoimmune and infectious diseases), age-related degenerative changes, and tumors are very common in clinical practice [3]. Although bone tissue possesses a certain degree of regenerative capacity, this ability is limited (commonly referred to as critical bone defects). For large bone defects that exceed the self-repair capability of bone tissue, bone grafting is typically required to achieve effective treatment outcomes [4]. Bone grafting primarily includes autologous and allogeneic bone grafts, as they possess fundamental properties that stimulate new bone growth, such as osteogenic capability, bone inductive activity, and bone conductivity [5]. However, autologous bone grafting has limitations related to donor site availability and the risk of donor site injury, while allogeneic bone grafting carries risks of immune rejection and secondary infections [6]. Furthermore, the treatment strategies for bone defects arising from different pathological conditions also exhibit significant variations [7].

The difficulties in traditional treatment strategies lie in controlling infection and reconstructing bone defects, with antibacterial treatment being a prerequisite for bone regeneration [8]. When bone defects are complicated by infection, it can lead to disturbances in microenvironment regulation, repeated infections, and even progressive exacerbation, which can hinder tissue repair. This condition can easily develop into severe osteomyelitis, bone necrosis, and even trigger septicemia, posing a threat to the patient’s life [9]. Intravenous administration often makes it difficult for local antibiotics to reach effective therapeutic concentrations. Prolonged systemic administration not only produces significant toxic side effects but also facilitates the development of resistant bacteria. Although antibiotic-impregnated spacers can enhance local drug concentrations and promote grafting through membrane induction, they require a second surgery approximately six weeks post-operation to remove the spacers [10, 11]. Bone transport techniques are an effective approach for treating bone defects; however, the treatment duration is lengthy, typically exceeding 10 months [12–14]. Additionally, traditional bone repair materials have been widely applied in clinical settings. However, their clinical performance remains unsatisfactory, such as poor mechanical properties, rapid absorption rates, and limited bone induction capabilities [15–17]. Therefore, there is an urgent need to develop new bone substitute materials to meet the clinical demands of bone repair. These materials must possess the following properties: osteogenic capacity, bone induction, bone conduction, biocompatibility, and biodegradability [18]. Osteogenic capacity refers to the ability of host and donor cells contained in the materials to synthesize new bone. Bone induction refers to the ability of materials to promote the differentiation of primitive pluripotent stem cells into mature osteoblasts. Bone conduction indicates the capacity of osteogenic and pre-osteogenic cells to attach to the implanted materials and migrate and grow within the three-dimensional space of the materials [19]. Furthermore, during the process of filling bone defects, new materials should also be able to mimic the structural, mechanical, and biological characteristics of natural bone, which may improve clinical outcomes for patients with bone defects and alleviate their suffering and economic burden in clinical practice [16, 20]. Consequently, the repair of inflammatory bone defects has remained a major challenge in orthopedic treatment, which not only leads to lasting physical and psychological harm for patients but also imposes a significant economic burden [21].

As mentioned earlier, if anti-infection therapy and the promotion of bone defect repair cannot be balanced, the therapeutic effect will be significantly reduced [22, 23]. Thus, dual-function biomaterials—combining antibacterial and osteogenic properties—offer promising solutions for treating infectious bone defects. These materials not only prevent infection at the defect site but also promote the regeneration of bone tissue, representing a significant advancement in bone defect repair [24–27]. For example, bioactive glasses (BGs) are multifunctional biomaterials that induce osteogenesis and angiogenesis [28]. A study by Kittipat et al. developed mesoporous bioactive glass composites loaded with antibacterial cinnamaldehyde (*CIN*), which demonstrated significant antibacterial activity against *Staphylococcus aureus* (*S. aureus*) and Escherichia coli (*E-coil*), while promoting bone tissue regeneration without affecting the proliferation of osteoblast-like *MG-63* cells [29]. Therefore, the intervention of dual-function biomaterials will open a new chapter in the repair strategy for infectious bone defects.

In general, the occurrence and healing of bone defects are closely related to inflammatory responses. Excessive acute inflammation caused by infection increases the risk of bone healing [30]. Therefore, to design more targeted dual-function biomaterials, it is essential to have a comprehensive understanding of the mechanisms underlying infectious bone defects and their local microenvironments. In this review, we will analyze the roles of different cells and inflammatory factors in the formation of new bone during the progression of bone defects, providing a theoretical basis for controlling infection and promoting osteogenic therapy. Based on this, we will summarize dual-function biomaterials with antibacterial and osteogenic properties and conduct an in-depth discussion. Finally, we will explore the development potential of future dual-function materials, offering innovative and feasible solutions for the treatment of infectious bone defects, and providing scientific and precise theoretical support and practical guidance for clinical treatment.

## Pathological processes of infectious bone defects

Bone healing is a complex process of reconstruction that occurs after a bone defect [31].It can be categorized into primary bone healing and secondary bone healing, with primary bone healing being relatively rare. Secondary bone healing consists of three overlapping stages: the inflammatory phase, the repair phase, and the remodeling phase, with the initial inflammatory phase being crucial for bone healing [32]. Each phase involves precise interactions among various immune cells, cytokines, and growth factors, ensuring a coordinated response to injury.

### Physiological processes in bone healing

**Fig. 1:**
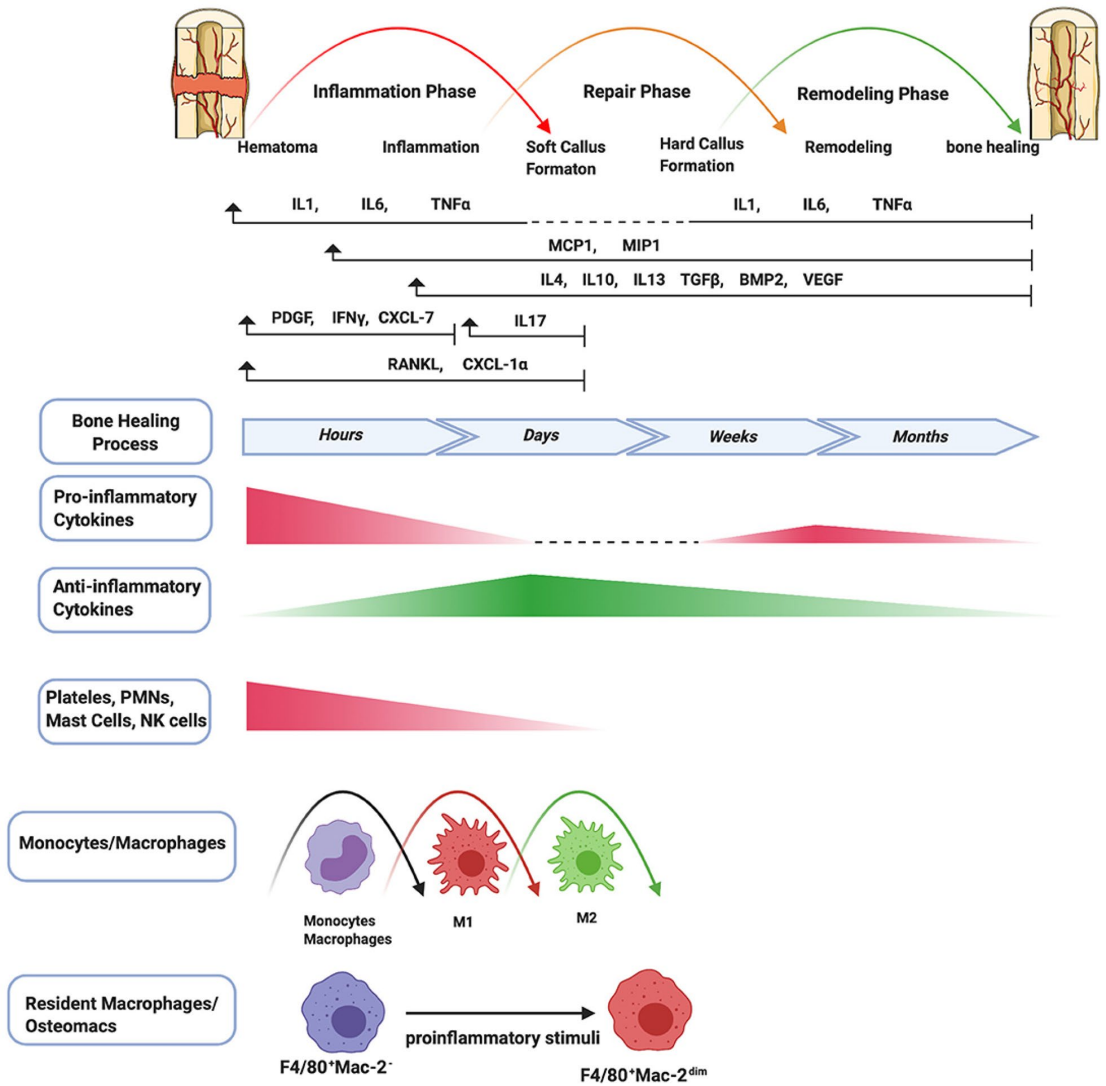
This describes the various stages of the bone healing process, as well as the expression patterns of related immune cells and cytokines/growth factors. Bone healing consists of three phases: inflammation, repair, and remodeling, which include six steps: hematoma formation, inflammatory response, soft (fibrocartilaginous) and hard callus formation, remodeling, and final healing. In the inflammatory phase, immune cells (like neutrophils, natural killer cells, mast cells, and platelets) are activated and release cytokines and chemokines to recruit monocytes and macrophages, essential for healing. Key pro-inflammatory cytokines such as *IL-1*, *IL-6*, and *TNF-α* play significant roles throughout the phases. *TNF-α* levels rise during the repair phase and remain elevated during remodeling, while the inflammatory response subsequently shifts to an anti-inflammatory state marked by *IL-4*, *IL-10*, and *IL-1*3, which are crucial for fracture healing. Copyright © 2020 Maruyama, Rhee, Utsunomiya, Zhang, Ueno, Yao and Goodman [33]

The process of bone healing can be further subdivided into six minor stages (Fig. 1). Once a bone defect occurs, the inflammatory response begins immediately. Hemorrhage beneath the periosteum and in the soft tissue leads to the formation of a hematoma. The microenvironment of the hematoma is initially characterized by localized hypoxia, acidity, and lower temperatures, with a high concentration of calcium and lactic acid [34]. During this process, the hematoma serves as a scaffold to recruit inflammatory cells and various factors to initiate a cascade reaction. Numerous inflammatory cytokines and growth factors are involved in this stage, including interleukin-1 (*IL-1*), interleukin-6 (*IL-6*), interleukin-11 (*IL-11*), and tumor necrosis factor-alpha (*TNF-α*), which promote the migration of inflammatory cells (such as neutrophils and macrophages) to the fracture site and induce angiogenesis [35, 36]. The secretion of these factors typically peaks around 24 h and is completed in about 7 days [37]. Among the interleukins, *IL-1* is the most important cytokine in bone healing and is primarily produced by macrophages. *IL-1* has functions such as inducing osteoblasts to produce *IL-6*, promoting the generation of bone healing tissue, and facilitating angiogenesis at the injury site (achieved by activating *IL-1RI* or *IL-1RII*) [38, 39]. *IL-6* is produced only during the acute phase, stimulating angiogenesis, the production of vascular endothelial growth factor (*VEGF*), and the differentiation of osteoblasts and osteoclasts [40–42]. Meanwhile, macrophages polarize towards the *M1* phenotype, and the immune response shifts to adaptive immunity, primarily characterized by the infiltration of lymphocytes. At the same time, fibroblasts also begin to gather at the site of injury [43]. Additionally, *TNF-α* has been demonstrated through multiple experiments to induce the osteogenic differentiation of mesenchymal stem cells (*MSCs*), which is crucial for subsequent bone healing [44]. Platelets and perivascular cells release several cytokines, such as platelet-derived growth factor (*PDGF*) and transforming growth factor-beta (*TGF-β*), which help promote angiogenesis and osteogenic differentiation [45]. During the resolution of inflammation, macrophages transform into *M2* phenotype under the induction of anti-inflammatory cytokines (*IL-4*, *IL-1*0, and *IL-13*).

To promote stable bone regeneration, it is crucial to recruit sufficient bone marrow-derived mesenchymal stem cells (*BMSCs*) to the defect site [46]. *BMSCs*, known for their pluripotency and self-renewal capabilities, play a pivotal role in osteogenesis [47, 48]. Factors such as *TNF-α* and stromal cell-derived factor 1 (*SDF-1*) have been shown to attract *BMSCs* to injury sites [49, 50].

Currently, a large number of studies have shown that *SDF-1* and its G protein-coupled receptor *CXCR-4* form an axis (*SDF-1/CXCR-4*), which is an important regulatory factor for the recruitment and homing of *BMSCs* to the injury site [51–55]. Under the influence of pro-inflammatory cytokines, anti-inflammatory cytokines, *TGF-β*, bone morphogenetic proteins (*BMP*), and growth factors (such as *VEGF*, *PDGF*), and fibroblast growth factor-2 (*FGF-2*), immune cells and *BMSCs* participate in the intercellular regulation to initiate the processes of osteogenesis and angiogenesis [56, 57].

Subsequently, the injury site enters the repair phase. The formation of granulation tissue involves two types of processes: intramembranous ossification and endochondral ossification [58]. The former is relatively rare and occurs on the periosteum, where *BMSCs* differentiate into osteoprogenitor cells, which then proliferate and differentiate into osteoblasts, directly forming woven bone. This process is continuous and is commonly observed in flat bones such as those in the skull and the clavicle [59]. The latter is the primary mechanism of bone repair, occurring within the endosteum and bone marrow, where *BMSCs* differentiate into chondrocytes and secrete cartilage matrix to form a cartilage template. Subsequently, chondrocytes undergo hypertrophic differentiation, leading to the mineralization of the surrounding matrix and the formation of cartilage granulation tissue. Ultimately, hypertrophic chondrocytes undergo apoptosis, resulting in vascular invasion and the migration of osteoblasts. Afterward, the cartilage matrix is transformed into bone matrix [60]. During this stage, the levels of pro-inflammatory factors such as *IL-1* and *IL-6* significantly decrease or are no longer detectable, while *TNF-α* initially decreases but later increases, meticulously regulating the proliferative activities of osteoblasts and shaping the overall healing response [50].

As the chondrocytes in the fracture granulation tissue proliferate, they become hypertrophic, and the ECM begins to calcify. During the period of 10 to 14 days, bone repair enters the stage of primary bone callus formation, with the formation of hard granulation tissue reaching its peak. The calcified cartilage is replaced by woven bone, making the granulation tissue stronger and mechanically stiffer [61]. Although the hard callus is a rigid structure that provides biomechanical stability, it does not fully restore the biomechanical properties of normal bone. To achieve complete restoration, the fracture healing process enters the second absorption phase, where the hard granulation tissue is remodeled into a layered bone structure with a central medullary cavity [62]. This phase is primarily regulated by *IL-1* and *TNF-α*, both of which exhibit high expression levels during this stage. Meanwhile, the content of the *BMP* family, represented by *BMP*−2, also increases, while the expression levels of most members of the *TGF-β* family decrease [63, 64]. During this period, the trabecular bone in the damaged area undergoes remodeling under the influence of mechanical stress. The combined action of osteoblasts and osteoclasts gradually eliminates the callus outside the stress axis, thereby facilitating bone healing. This process may take several years to achieve complete regeneration of the skeleton; however, in younger patients, the healing process is typically accelerated [65]. The original callus is gradually replaced by solid lamellar bone, completing the creeping substitution process of the new bone. In addition, growth factors such as epidermal growth factor (*EGF*) and fibroblast growth factor (*FGF*) also promote angiogenesis and the maturation of chondrocytes during bone repair and reconstruction [33]. Successful bone remodeling requires adequate blood supply and gradually increasing mechanical stability. If these conditions are not met, atrophic nonunion may occur [66, 67]. Conversely, when the vascular condition is good but fixation is unstable, it can lead to hypertrophic nonunion or pseudarthrosis [68].

As mentioned above, inflammation is the critical first step in bone healing. The absence or suppression of acute inflammation can lead to impaired bone healing [69–71].Research on *MIF* mice (macrophage migration inhibitory factor gene-deficient mice, *MIF KO*) and immune-compromised *NOD/scid-IL2Rγc* null mice has shown that deficiencies in immune function can impair bone healing, highlighting the importance of an intact immune response for effective bone repair [72–76]. Additionally, multiple pro-inflammatory cytokines and chemokines, such as *IL-6*, *IL-17A*, and *TNF-α*, play crucial roles in the complex cascade of bone fracture healing [77–79]. For example, *IL-6* signaling is involved in immune cell recruitment, angiogenesis, endochondral ossification, and granulation tissue remodeling [80]. *IL-17A* enhances the activity of osteoblasts involved in bone tissue formation, while *TNF-α* has a biphasic secretion pattern and can enhance the activation of *BMSCs *[49, 81]. Furthermore, prostaglandins like Prostaglandin E2 (*PGE2*) and non-steroidal anti-inflammatory drugs (*NSAIDs*) that block *COX-2* can adversely affect bone healing [82–85]. Glucocorticoids, although powerful anti-inflammatory agents, can delay bone repair and cause osteoporosis [86, 87]. These findings underscore the crucial role of inflammation in bone healing.

### Microenvironment of Infectious Bone Fractures

The microenvironment of infectious bone fractures is complex and often leads to an intense inflammatory response that disrupts normal bone healing [88, 89]. This section will discuss the relationship between local inflammation and bone regeneration, as well as analyze the effects of infection on the ECM, osteoblasts, and osteoclasts.

**Fig. 2:**
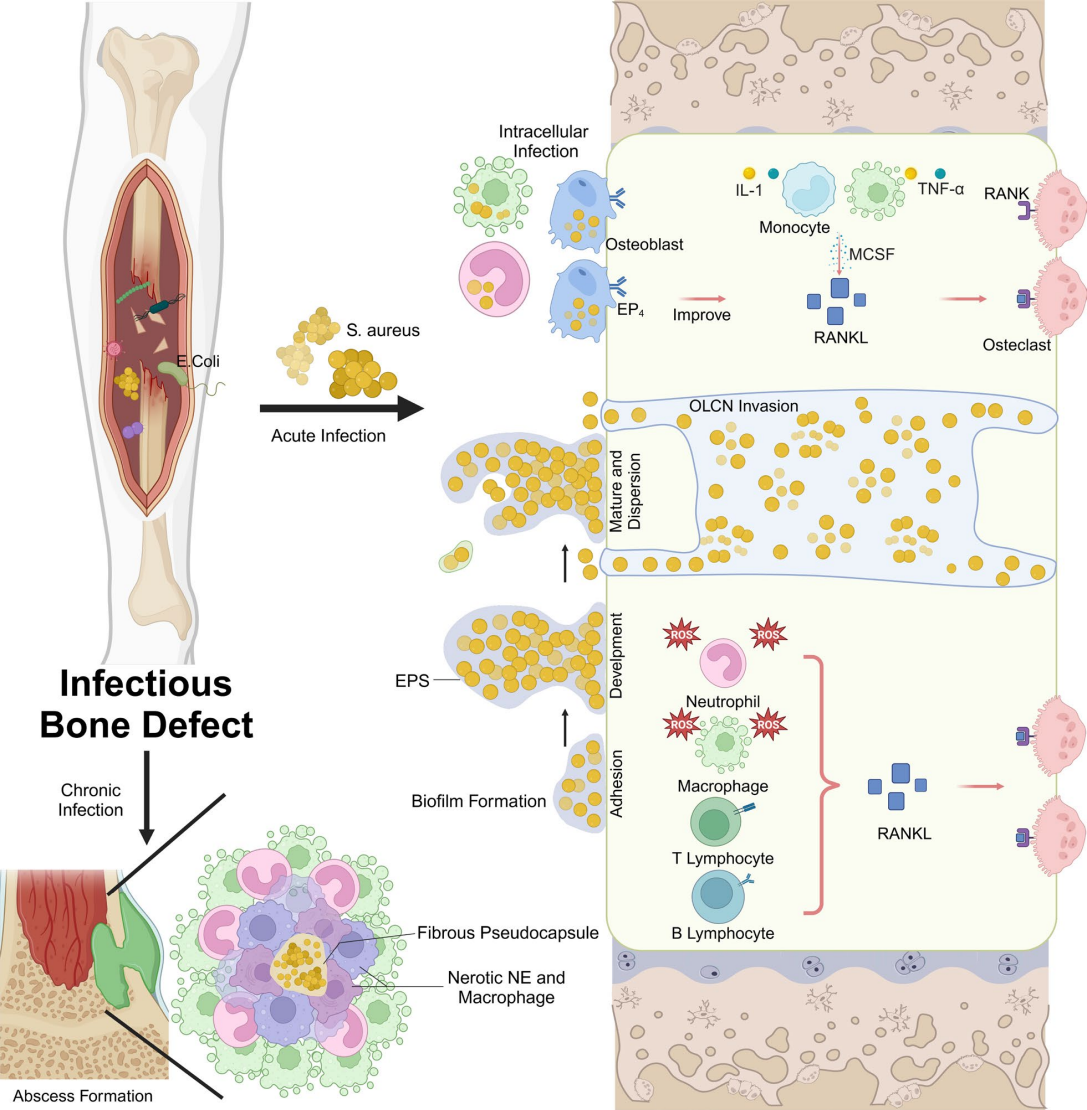
Schematic diagram of the key pathogenic mechanisms of infectious microorganisms in bone defects and their partial immune regulatory effects in the bone marrow cavity. (Created with BioRender.com)

The most common bacterium responsible for bone infections is *S. aureus*. Its primary infection mechanisms include intracellular infection, osteocyte lacunar canalicular network (OLCN) invasion, biofilm formation, and abscess formation (Fig. 2) [90]. This bacterium can proliferate in various cell types, including but not limited to macrophages, keratinocytes, epithelial cells, endothelial cells, osteoclasts, osteoblasts, and bone cells [91]. After invading the osteoblasts, fibronectin connects the fibronectin-binding proteins A or B (*FnBPA* or *FnBPB*) on the surface of *S. aureus* cells with the *α5β1* integrin on osteoblasts. This interaction facilitates bacterial internalization [92]. Since osteoblasts are not immune cells, *S. aureus* can survive for a long time within these cells, rendering internalized cells less sensitive and allowing them to evade clearance by the immune system and killing by antibiotics. The infected cells become a reservoir that subsequently releases pathogens, continuing to infect other cells [93, 94]. Research has indicated that osteoblasts can secrete a certain number of antimicrobial peptides; however, without proactive and effective treatment, the damaged area may progress to a chronic infection [95, 96].

Although *S. aureus* has a diameter of approximately 1 μm, it can alter its size to 100–600 nm to invade the OLCN, which has a diameter of 0.5 μm [97–101]. This submicron-level invasiveness allows the bacteria to evade immune cells and survive long-term in the bone. Additionally, high-dose antibiotics struggle to achieve effective concentrations within the OLCN, highlighting the need for biomaterials that can resist bacterial invasion.

Biofilm formation is another significant challenge *S. aureus* adheres to bone surfaces using cell wall proteins, adhesins, eDNA, and microbial surface components recognizing adhesive matrix molecules (MSCRAMM). These components interact with proteins and polysaccharides in the bone cell ECM, such as type I collagen and osteopontin, forming binding sites [102–105]. The bacteria then produce extracellular polymeric substances (EPS) that protect and promote their growth, leading to a dense biofilm structure that shields them from antimicrobial agents and immune cells [106, 107]. Therefore, developing materials that can inhibit and eliminate biofilms is essential.

Furthermore, EPS degradation allows free bacteria to colonize new areas and form new biofilms [108]. Persistent *S. aureus* infections can lead to staphylococcal abscess communities (SACs), which affect the bone marrow and surrounding soft tissues of implants [109]. The bacteria form a fibrin pseudo-capsule using coagulase (*CoA*) and von Willebrand factor-binding protein (*vWbp*), attracting immune cells and causing their necrosis, thus creating a barrier that prevents new immune cells from entering and forming a bacterial sanctuary [110–112]. Once an abscess forms, the probability of spontaneous recovery without targeted antibacterial strategies is very low [113]. Developing biomaterials that can disrupt the fibrin pseudo-capsule is a promising approach for treating abscesses.

The balance between bone regeneration and bone resorption is crucial for maintaining local bone mass in the area of bone defects and for reconstructing normal bone tissue structure [114]. At the microscopic level, infectious bone defects exhibit elevated levels of inflammatory cells compared to sterile bone injuries, with various pro-inflammatory factors such as *IL-1β* significantly increased [115]. These factors can enhance the activity of osteoclasts, leading to increased bone resorption, while inhibiting the function of osteoblasts, thereby hindering the biological processes of bone regeneration [116]. Clearly elucidating the molecular interactions between pathogens, immune cells, and various bone cells within the infectious microenvironment, as well as the mechanisms of bone regeneration, holds significant research importance and clinical value. The influence of immune cells on bone regeneration in the infectious microenvironment is particularly noteworthy.

Neutrophils (*NE*) are highly active phagocytic cells that rapidly accumulate during the early stages of pathogen invasion. They migrate to the site of infection through various chemokines, moving through the gaps between endothelial cells to form neutrophil extracellular traps (NETs), where they perform functions such as phagocytosis, reactive oxygen species (*ROS*) production, and degradation of pathogens by granzyme [117]. However, the excessive accumulation of NE can often lead to side effects such as a high-oxygen environment induced by *ROS*, resulting in cytotoxicity, bone tissue damage, and bone loss [118, 119]. Additionally, NE expresses elevated levels of receptor activator of nuclear factor-κB ligand (*RANKL*) in response to stimuli from pathogenic virulence factors like LPS. By binding to the receptor activator of nuclear factor-κB (*RANK*) on pre-osteoclasts, *RANKL* induces the differentiation and maturation of osteoclasts, thereby promoting bone resorption [120]. Besides *NE*, macrophages, activated T cells, and B cells are also capable of releasing substantial amounts of *RANK*L in vivo, further inducing bone resorption and bone loss [121].

In addition to NE, macrophages also play a role in the early stages of infection. As mentioned earlier, macrophages are a major source of cytokines and release large amounts of *TNF-α* and *IL-1* to activate endothelial cells and lymphocytes to gather at the site of infection. At the same time, macrophages induce endothelial cells to form P-selectin, recruiting NE to the infection site to exert immune functions, while also performing phagocytosis and releasing *ROS* to combat the infection [122–124]. However, prolonged inflammation mediated by macrophages can inhibit the formation and maturation of new blood vessels, reduce the rate of bone healing, and suppress the differentiation of osteoblasts. Additionally, the various cytokines released may increase the production of *RANK*L, leading to the side effect of bone resorption [125]. Among these, sustained high levels of *TNF-α* can inhibit the *BMP*−2 signaling pathway by promoting the ubiquitination and proteasomal degradation of Smad1 and runt-related transcription factor 2 (*Runx-2*), thereby hindering the bone regeneration process [126, 127].

Osteoclasts differentiate from hematopoietic mononuclear cells/macrophages and are regulated by macrophage colony stimulating factor (*M-CSF*) and *RANKL*. *M-CSF* can also promote the binding of *RANKL* to *RANK* on the surface of osteoclasts, thereby increasing their sensitivity and facilitating osteoclast differentiation [128, 129]. Osteoblasts infected by pathogens can directly or indirectly promote the secretion of *RANKL* through the *EP4* receptor, while simultaneously downregulating the expression of *OPG*, further promoting the generation of osteoclasts [130, 131].

In summary, the infectious microenvironment creates a persistent inflammatory state, enhancing osteoclastic activity and impeding osteogenesis, which collectively disrupts bone healing. Addressing the mechanisms underlying this altered environment is critical for developing effective treatments. Novel biomaterials that can regulate inflammation, disrupt biofilms, and promote osteogenesis represent promising therapeutic strategies for managing infectious bone fractures.

## Design concepts for bifunctional biomaterials

In simple terms, the ideal treatment plan for infectious bone defects is a combination of rapid infection control and bone tissue repair. Consequently, the ideal biomaterials should be designed to release antimicrobial agents rapidly or exert bactericidal effects in the initial stage. They need to maintain a high concentration for a sufficient duration to effectively control and suppress bacterial proliferation. This is of great significance as uncontrolled infection can lead to further bone destruction and delay the healing process. Importantly, this process should not compromise bone regeneration. On the contrary, it should create a favorable environment for bone repair. Furthermore, these materials should also maintain high biocompatibility and mechanical strength. The high biocompatibility ensures that there are no adverse immune responses, and the mechanical strength provides support for the newly formed bone tissue. This enables osteoblast migration and differentiation on the biomaterial scaffold, promoting the integration of the material with the host bone and the formation of new bone tissue [132–134].

### Selection of antimicrobial materials

For infectious bone defects, antibiotics such as vancomycin (*VAN*), rifampicin, amoxicillin, and levofloxacin remain effective first-line antimicrobial agents. However, they also have some drawbacks, including dose dependence, bacterial resistance arising from high-dose usage, and potential effects on systemic and local cells [135, 136]. In recent years, several new treatments for bone infection, in addition to traditional antibiotics, have also drawn attention.

#### Inorganic materials

Most inorganic materials, excluding metals such as Ag^+^, Zn^2^⁺, Cu^2^⁺, or La^3^⁺, that lack antibiotics or functional components generally do not possess antimicrobial properties. They are primarily used to promote bone formation or serve as scaffolding (see Section "Inorganic materials" for details) [137].

Inorganic metal particles represented by gold, silver, zinc, and copper significantly affect bacteria due to their unique physicochemical properties, such as size, zeta potential, surface morphology, and structure [138]. The intermolecular forces between metal particles, such as receptor-ligand interactions, electrostatic interactions, and van der Waals forces, can act on bacterial cell membranes. When they bind with cations, they cause a change in membrane charge, thus breaching the protective biofilm of the bacteria, leading to membrane rupture and bacterial death [139]. Additionally, metal particles can damage the proteins and DNA inside bacteria, affecting various metabolic pathways and hindering biosynthesis, ultimately resulting in bacterial death [140]. Furthermore, metal particles can convert hydrogen peroxide into highly toxic hydroxyl radicals through the Fenton reaction, leading to the substantial production of *ROS* and subsequently inducing bacterial death [141].

However, metals are a double-edged sword; while they can kill bacteria, they also exhibit cytotoxicity [142]. Therefore, many researchers have modified metal particles by constructing composite scaffold systems through methods such as blending with scaffold substrates, coating preparation, and material modification grafting. These approaches not only reduce cytotoxicity but also optimize the performance of metal particles, significantly enhancing the selectivity of the materials [143]. For example, Zhang et al. prepared PLA coatings containing different concentrations of Cu^2^⁺ on titanium sheets using a dip-coating method. This coating significantly reduced the biotoxicity of Cu^2^⁺ to *BMSCs*. At the same time, the Cu^2^⁺ coating promoted healing at the fracture site while significantly reducing infections, achieving a balance among antibacterial effects, bone regeneration, and cellular compatibility. Within 28 days post-surgery, the levels of Cu^2^⁺ in the blood returned to normal [144]. A study applied nano zinc oxide to coat 3D-printed 3Y-ZrO₂ scaffolds. These scaffolds demonstrated high biocompatibility and excellent antibacterial activity in both in vitro and in vivo experiments [145].

#### Organic materials

Organic antimicrobial materials are substances composed of organic compounds that possess inherent or potential antimicrobial properties. These materials typically include natural organic antimicrobial materials and synthetic organic antimicrobial materials [146].

When it comes to natural organic antimicrobial materials, chitosan (CS) is the most classic natural cationic polysaccharide. It is mainly obtained through the deacetylation of chitin, which is derived from the exoskeleton of crustaceans. CS has advantages such as non-toxicity, biodegradability, broad-spectrum antibacterial properties, adsorption capacity, and good biocompatibility [132, 147, 148]. Compared to common antimicrobial agents, CS exhibits unique characteristics, such as broad-spectrum efficacy, high bactericidal rates, and effective inhibition of the growth and reproduction of bacteria and fungi [149]. The main mechanisms of antimicrobial action are as follows:1. Cationic CS molecules react with negatively charged bacterial molecules on the cell surface, altering the cell's permeability, inhibiting bacterial metabolism, and leading to bacterial death [150]. 2. CS can penetrate bacterial cells and bind to DNA, thereby inhibiting protein synthesis related to gene expression in bacteria. Additionally, it can adsorb negatively charged substrates within microbial cells, disrupting their physiological activities and resulting in cell death [151]. 3. CS can inhibit the absorption of alkaline elements necessary for microbial growth through a metal chelation mechanism, while binding to metal ions required by the microbes to achieve antibacterial effects [152]. 4. High molecular weight CS can adhere to cell walls, reducing the absorption of nutrients from the extracellular environment [153].

Due to the excellent properties of CS, various modification schemes have been extensively studied. For instance, the antibacterial effect of nanoscale CS particles has been significantly enhanced through chemical modification, which increases surface charge density and hydrophobicity, thereby improving antibacterial activity [154–157]. CS loaded with antibiotics has demonstrated synergistic antibacterial effects. Kimna et al. constructed a chitosan-montmorillonite nanoclay material loaded with *VAN* and gentamicin (GM), achieving encapsulation rates of up to 98% for both VC and GM, with a release duration of up to 30 days. This composite exhibited strong antibacterial activity against gram-positive (G^+^) *S. aureus* and gram-negative (G^−^) *E-coil*, while showing no cytotoxic effects on normal cells [158]. In addition to traditional antibiotics, metal chelation, coatings for implant scaffolds, and synthetic hydrogel scaffolds also exhibit good antibacterial effects [159–161]. For example, Yang et al. incorporated graphene oxide/copper nano derivative (GO/Cu) into CS/hyaluronic acid (HA) scaffolds. The constructed composite material demonstrated satisfactory in vivo anti-infection performance by damaging the bacterial membrane, increasing *ROS* production, and disrupting key enzyme metabolism, which reduced inflammation and maintained acceptable biosafety. Additionally, the GO/Cu material (mass ratio = 2:1) exhibited enhanced osteogenic differentiation and promoted bone healing [162].

HA is a linear glycosaminoglycan that is widely used in fields such as trauma, orthopedics, and joint replacement surgery. It primarily exists in the ECM and possesses good biocompatibility, hydrophilicity, and lubricating properties [163]. HA can act as an antibacterial material by disrupting bacterial integrity or serve as a carrier for drugs and coatings for implants [164–166]. For instance, Valverde et al. immobilized HA and CS on the surface of Ti-6Al − 4 V alloy to form polyelectrolyte multilayers (PEMs), which exhibited good efficacy against *S. aureus* [167].

Antimicrobial peptides (*AMPs*), also known as host defense peptides, are components of the innate immune system and exhibit strong antibacterial properties against various microorganisms [168]. Their antibacterial mechanisms mainly include: 1. Disrupting bacterial signal transduction and interfering with biofilm formation to reduce attachment capability; 2. Blocking the synthesis of products related to bacterial starvation to inhibit biofilm formation; 3. Suppressing the expression of DNA related to bacterial starvation products, thereby fundamentally inhibiting biofilm formation [169]. However, *AMPs* have a short half-life in vivo and are prone to degradation by proteases or binding with plasma proteins, thereby affecting their biological activity. Consequently, researchers typically combine *AMPs* with bioactive materials, with strategies mainly including physical mixing and encapsulation, nanoparticle construction, and biocoating, to improve these drawbacks [170, 171]. He et al. constructed a mineralized collagen scaffold loaded with two *AMPs* (*Pac-525* and *KSL-W*) using poly (lactic-co-glycolic acid) (PLGA) [172]. In antibacterial experiments, this scaffold exhibited excellent antibacterial efficacy for the long-term inhibition of *S. aureus* and *E-coil*. This is due to the interaction of the antimicrobial peptide with the bacterial cell membrane. *Pac-525* and *KSL-W* disrupt the membrane integrity through electrostatic interactions, leading to bacterial death. Cell proliferation and alkaline phosphatase assays also demonstrated its good osteogenic capacity. The mineralized collagen component of the scaffold provided excellent osteoconductivity and osteoinductivity, promoting cell adhesion, proliferation, and osteogenic differentiation. The porous structure of the scaffold facilitated cell infiltration and nutrient transport. The mechanical properties of the scaffold were sufficient to support bone regeneration, and the PLGA microspheres ensured the sustained release of antimicrobial peptides, maintaining an effective concentration for long-term antibacterial activity. This study provided a broad prospect for the treatment of infectious bone defects. To address potential resistance, protein antibacterial materials inspired by *AMPs* have been developed, consisting of other amino acids linked at different positions via peptide bonds to disrupt the structure and function of bacteria [173].

Most synthetic organic materials are typically not utilized as antibacterial agents in the treatment of infectious bone defects [174]. Well-researched antibacterial materials can be categorized into over twenty types based on their molecular structure, including quaternary ammonium salts, biguanides, alcohol aldehyde esters, ethers, phenols, and imidazoles. Among these, antibacterial materials from the quaternary ammonium salt category are used more extensively [175]. Polymethyl methacrylate (PMMA) bone cement is a widely used synthetic organic bone repair material that has been clinically applied extensively over the past few decades. Various antibiotics, such as gentamicin and *VAN*, have been incorporated into PMMA to address bacterial infections [176, 177]. To extend the antibacterial spectrum to drug-resistant bacteria and viruses, and to enhance the release of antibacterial agents, Wang et al. developed a quaternary ammonium salt-modified antibacterial PMMA bone cement. This material exhibits high mechanical strength and sustained release of antibacterial agents, showing good antibacterial efficacy against both G^+^ and G^−^ bacteria, with stronger antibacterial activity against G^−^ bacteria [178]. However, in the treatment of infectious bone defects, quaternary ammonium salts are typically used to modify CS to enhance its antibacterial properties [179]. Therefore, these materials have significant research potential.

### Selection of bone repair promoting materials

#### Inorganic materials

Bone tissue contains a variety of metal elements that play an indispensable role in bone regeneration at both molecular and cellular levels [180]. Consequently, metal particles may provide more direct signals for bone regeneration. For example, ions such as Ca^2^⁺, Mg^2^⁺, Sr^2^⁺, Zn^2^⁺, Li⁺, Mn^2^⁺, Cu^2^⁺, Co^2^⁺, Ce^3^⁺, Fe^3^⁺, and Ag⁺ are used in bone regeneration. These elements regulate multiple metabolic processes, including the differentiation and activity of osteoblasts and osteoclasts, bone mineralization, vascular regeneration, and immune modulation [1]. Different metal ions exert distinct effects in these areas.

The most abundant Ca^2^⁺ in the bones provides strength and supports movement, while also participating in various signaling pathways. Optimal fluid concentrations can stimulate the proliferation, migration, and mineralization of osteoblasts, enhance their adhesion capacity, and induce macrophages to polarize towards the *M2* phenotype [181–183]. Mg^2^⁺ is the fourth most abundant mineral in the human body, primarily found in the bones, and is a fundamental element of bone tissue [184]. It plays a role in the following three areas: (1) Promoting the osteogenic differentiation and adhesion of *BMSCs*, facilitating the migration of osteoblasts and inhibiting the generation of osteoclasts [185–187]; (2) Inducing macrophages to polarize towards the *M2* phenotype, eliminating oxidative stress in LPS-induced macrophages [188, 189]; and (3) Promoting the angiogenic differentiation of *BMSCs* [190]. Sr^2^⁺ is primarily found in areas of active growth and regeneration of fresh trabecular bone, where it functions to prevent bone resorption and stimulate bone formation. It promotes vascularization of bone tissue by activating the *PDGF-BB/PI3K/AKT* signaling pathway [191, 192]. Additionally, Sr^2^⁺ and its modified materials exhibit anti-inflammatory properties, capable of regulating the polarization of neutrophils, leading to the production of anti-inflammatory cytokines, and thereby directly or indirectly inducing macrophages to polarize towards the *M2* phenotype [193, 194]. Recent studies have found that Sr^2^⁺ significantly inhibits the growth of *E. coli* and partially suppresses the growth of *S. aureus*, indicating its potential in the treatment of infectious bone defects [195]. Although Zn^2^⁺ constitutes only 30% of the bone, it is an important cofactor for the stability of bone microstructure and the remodeling of bone proteins, playing a critical role in bone growth and stability [196]. It can promote the adhesion, proliferation, and differentiation of osteoblasts, inhibit osteoclasts, regulate the differentiation of inflammatory cells, induce macrophages to polarize towards the *M2* phenotype, and facilitate angiogenesis, while also exhibiting excellent antibacterial properties [197–200]. However, it is important to note that high concentrations of Zn^2^⁺ may enhance the activity of osteoclasts [201]. Other metal ions also play significant roles in bone metabolism. In the application of tissue engineering for promoting bone repair, metal ions are often incorporated into biomaterials to improve their release kinetics, optimize the regulation of bone metabolism, and enhance biocompatibility.

In addition to metals, bioceramics represented by calcium phosphate (CaP) bone cement, hydroxyapatite (HAp), and α/β-tricalcium phosphate (α/β-TCP) are widely used in the preparation of multifunctional scaffold systems for infectious bone defects [202–204]. Bioceramic scaffolds are fabricated as follows: The powders are shaped into desired forms using techniques like dry pressing, slip casting, or 3D printing. The bodies are then subjected to high-temperature sintering to enhance density and mechanical strength, with sintering parameters significantly influencing the final properties. Finally, surface treatments or coatings are applied to optimize bioactivity, making them suitable for a wide range of applications in orthopedics, dentistry, and cardiovascular medicine [205]. Although they have certain shortcomings in antibacterial performance, their composition is highly similar to the inorganic salts found in human bone tissue. In addition to good biocompatibility, they possess high mechanical strength, which is often lacking in organic materials. Therefore, these materials can serve as carriers for antibacterial agents and bone fillers, combined with various antibacterial mechanisms, showing bright prospects in bone tissue scaffold applications [137].

Wu et al. modified calcium phosphate cements (CPCs) to construct an injectable CPC-chitosan scaffold with strong mechanical properties. This scaffold, combined with penicillin-alginate microspheres, can be completely injected with lower injection force. In vitro experiments demonstrated its effective antibacterial function against *S. aureus* infections, and it possesses strength comparable to that of trabecular bone, allowing for good growth of Human Umbilical Cord Mesenchymal Stem Cells (*hUCMSCs*) within the scaffold. If further validated with in vivo experiments using animal models, this scaffold would hold significant clinical translation potential [206].

A study functionalized magnesium hydroxyapatite (Mg-HAp) with silver nanoparticles (AgNPs) to construct an Ag-Mg-HAp scaffold, which demonstrated good antibacterial properties against *E. coli* and *S. aureus*. However, in the same living environment, the proliferation of human adipose-derived stem cells (*hADSCs*) decreased by 90% on day 24. Consequently, researchers need to further optimize the cytotoxicity [207]. β-TCP is an important inorganic component of natural bone tissue, exhibiting excellent bioactivity, osteoconductivity, and osteoinductivity. Compared to PMMA, it can degrade in vivo, providing appropriate space for bone repair [208, 209]. Liu et al. improved the toughness, brittleness, and high elastic modulus deficiencies of the scaffold material using gelatin and encapsulated CS microspheres containing gentamicin (GM). This material not only has good antibacterial activity against *S. aureus* but also shows excellent strength and osteoconductivity at different stages of bone repair in in vivo infection models [210].

#### Organic materials

CS exhibits both antibacterial properties and the capability to promote bone regeneration. This is because it is similar to glycosaminoglycans in the natural ECM, creating a favorable local microenvironment for cell growth and supporting the proliferation, differentiation, and mineralization of osteoblasts [211]. Studies have found that the higher the deacetylation degree (DD) of CS, which indicates the number of amino groups, the stronger the cell adhesion capacity. Additionally, the cations carried by CS can enhance cell activity through methods such as electrical stimulation. Research indicates that both osteoblasts and *MSCs* can proliferate and differentiate well in a CS environment [132, 212]. Due to the excellent compatibility of CS with other materials, it can synergistically enhance bone generation. Oftadeh et al. merged HAp with CS and found that various osteogenic markers, such as osteocalcin, alkaline phosphatase, osteonectin, and *Runx2*, showed significantly upregulated transcription levels in cells, further enhancing osteogenic capability [213]. Combinations with metal ions, graphene oxide (GO), bioglass, and bioactive substances such as growth factors and *BMP*−2 also demonstrate considerable potential in bone defect repair [214–219].

Collagen and gelatin possess ideal characteristics for applications in bone tissue engineering and are one of the main protein components in natural bone. Their amino acid sequences contain adhesive ligands-arginine-glycine-aspartic acid (RGD)—which facilitate the attachment of various cells [220, 221]. Furthermore, these components exhibit excellent biocompatibility and can be degraded by recipient enzymes, making them excellent biological carriers for other components [222–224]. Alginate is also a natural polymer derived from algae, known for its good biocompatibility and plasticity. The addition of RGD can promote cell adhesion or confer specific functions through other factors and functional groups [225, 226].

Synthetic organic materials usually do not possess antibacterial properties. However, compared with natural organic materials, they offer more possibilities for chemical modifications and molecular changes, making them excellent carriers for drugs or antibacterial materials. In the field of biological carrier treatment for bone infections, poly lactic-co-glycolic acid (PLGA) is a classic synthetic organic material. It is a copolymer of polylactic acid (PLA) and polyglycolic acid (PGA), featuring excellent biocompatibility and biodegradability, with adjustable mechanical properties, thus offering significant advantages as a carrier for the in vivo delivery of antibiotics [227, 228]. Joaquin et al. developed a PLGA microsphere modified with daptomycin and *VAN* in Palacos R cement, which demonstrated good antibacterial properties in a model of infectious bone defects, while preserving the normal structure of the surrounding bone and reducing damage to the bone tissue [229]. Materials such as PMMA, polycaprolactone (PCL), and polyetheretherketone (PEEK) also play important roles in the treatment of infectious bone defects [230–232]. Next, we will introduce several types of composite materials.

## Bifunctional biomaterials

**Fig. 3:**
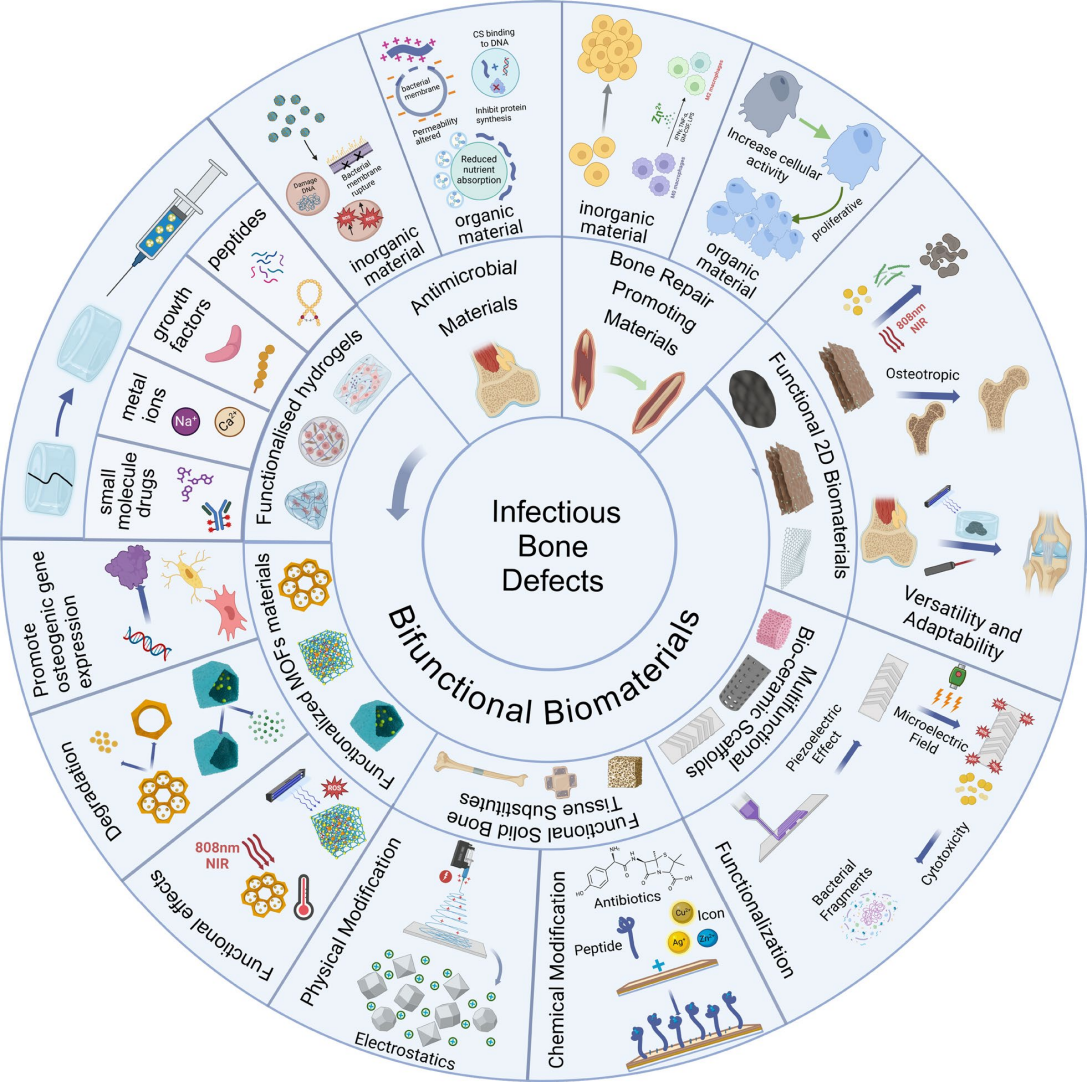
Schematic diagram of strategies and mechanisms involving partial bifunctional materials for the treatment of infectious bone defects (Created with BioRender.com)

Composite biomaterials present promising biomimetic solutions that effectively address significant challenges within this field, owing to their performance often surpassing that of their individual constituents. The main strategies include incorporating nanoparticles as fillers into biodegradable biological carriers or constructing polymers using covalent bonds and other methods [233]. Many of the studies mentioned earlier are based on these innovative findings. Bioactive ceramics and glass particles are capable of enhancing the mechanical strength of polymers while concurrently promoting bone tissue regeneration [234]. Additionally, some bioactive materials can provide polymers with extra functions, such as shape memory (for example, injectable and self-healing hydrogels) and photothermal effects (like black phosphorus, BP) (Fig. 3) [235–237].

### Hydrogels with antibacterial function for promoting bone defect repair

Hydrogels are polymers with a three-dimensional network structure that can be formed into various shapes and sizes [238]. Additionally, these materials contain a high degree of hydrophilic groups, allowing them to effectively capture and retain large amounts of moisture or biological fluids [239]. Simultaneously, hydrogels are similar in morphology and function to natural extracellular matrices, providing a favorable environment for cells to support nutrient delivery, cell proliferation, and differentiation, while minimizing damage to biological tissues during this process [240]. In addition to utilizing the inherent properties of hydrogels to repair infectious bone defects, they can also promote therapeutic effects by loading peptides, growth factors, metal ions, or small molecule drugs (Table 1).

**Table 1:** A partial composite hydrogel strategy for treating infectious bone defects

Categories	Components	Strategy	Assessments of infection	Assessments of bone healing	Year
Gelatin methacrylate (GelMA)	β-TCP; Sodium alginate (Alg); Strontium chloride (Sr^2+^); MXenes (Ti_3_C_2_)	Photothermal Therapy (PTT) Osteogenic Stimulation	*Vitro*: Significant inhibition against *S. aureus* and *E. coli* (Viability Reduction: 1.4% and 1.1%)	*Vitro*: mRNA expression levels of *Runx2*, *ALP*, and *OCN*↑; *ALP* activities and protein expression levels of *ALP* and *OPN*↑; *Vivo*: Micro-CT showed Bone volume/Tissue volume (BV/TV) ratios↑; H&E staining: Direct connection of new bone with host bone; Immunohistochemical staining: *ALP* and *OCN*↑	2022[241]
GelMA	N-acryloyl glycinamide (NAGA); Molybdenum Disulfide (MoS_2_); Gadolinium (Gd)	PTT; Tumor Ablation	*Vitro*: Effective antibacterial activity against *S. aureus* and *E. coli* *Vivo*: Bacterial infections and inflammation↓ (*S. aureus*-infected rat tibia defects)	Osteogenic Differentiation: Gd^3+^ ions promoted osteogenic differentiation of rat osteoblasts (ROBs); Bone Regeneration: 1. Micro-CT and histological analysis: Significant New bone formation; 2. Quantitative data: BV/TV (11.83%); 3. Fluorochrome-labeling: 6.85% (12 weeks)	2023[242]
GelMA	Copper ion-modified germanium phosphorus nanosheets (GeP@Cu); Gd	Conductivity and Biodegradability	Antibacterial rates of 95.51% ± 2.15% (*E. coli*) and 94.28% ± 4.16% (*S. aureus*); *ROS* Generation	1.Osteogenic Differentiation of *BMSCs*, *ALP* activity and mineralized matrix formation↑; 2. Neurogenic Differentiation and Angiogenesis↑; 3. Micro-CT: BV/TV and trabecular number (*Tb.N*) after 12 weeks↑	2023[243]
GelMA	Proanthocyanidin; Amikacin; Boronate Complexes	Dual Delivery System; pH and *ROS* Responsiveness;	Antibacterial rates of *S. aureus* and *E. coli* were 87.4% and 78.4%; Bacterial Morphology: Damaged bacterial morphology	*ALP* activity and gene expression levels of *Alp, Runx2*, *OCN, an*d *Col1*↑	2024[244]
Carboxymethyl Chitosan (CMCS); GelMA	*BMP*−2; PLGA; GO; Antisense yycF (ASyycF)	Photo-crosslinking; Spring Structure	Antibacterial property of composite hydrogel 20 times higher; Biofilm formation (*S. aureus*) ↓;	*Vitro*: *ALP* (7 days) and Alizarin Red staining (14 days) ↑; *Vivo*: 1. Large amounts of new osteotylus formation and effective infection control (8 weeks); 2. Micro-CT: nearly complete healing of the bone defect; 3. Histopathological Analysis: Deposition of collagen and new bone formation	2023[245]
CS Sulfated-(SCS)	Oxidized HA; β-Sodium Glycerophosphate (β-GP); Cu-Sr Doped Mesoporous Bioactive Glass (CuSrMBG)	Dual-Network Formation	1.*Vitro*: Number of *E. coli* and *S. aureus*↓ (> 95%); 2.*M1* → *M2*	Osteogenic Differentiation: *ALP* and mineral deposition↑; Bone Regeneration: BV/TV, bone mineral density (*BMD*), trabecular structure↑	2024[246]
Aldehyde hyaluronic acid (AHA); Carboxymeth-yl chitosan (NOCC)	PCL; Van; Mesoporous silica nanoparticles (MSNs); Fingolimod (FTY720)	Dual-Drug Delivery; Structural Support	*Vitro*: Scaffolds showed strong growth inhibition against *S. aureus* and *E. coli*	1.The scaffold supported *BMSCs* growth; 2. *ALP* activity and calcium nodule formation↑ 3. *Vivo*: BV/TV and Trabecular Thickness (Tb.Th)↑	2023[247]
Gellan Gum (GG)	HAp; Chlorhexidine	Injectable Delivery System	Significant inhibition of *S. aureus* biofilm formation was observed at a concentration of 50 mg/mL; Lower bacterial colony counts in infectious bone defects	New Bone Formation at 4,8 weeks↑; Micro-CT: Value of BV/TV were 0.32 ± 0.11 (4 weeks) and 0.49 ± 0.13 (8 weeks) Histological Analysis: Defect areas largely consisted of blue-stained newly formed woven bone, indicating active bone regeneration	2022[248]
Polyethylene Glycol Diacrylate (PEGDA)	Copper (Cu)-Strontium (Sr) Peroxide Nanoparticles (CSp NPs)	Dual Metal Peroxides; pH-Responsive Release (Ph = 5.5: Antibacterial activity pH = 7.4: Osteogenesis)	*Vitro*: IC50 of *S. aureus* was 25 ± 0.796 μg/mL; A killing rate of 99.94% at 50 μg/mL *Vivo*:	Proliferation and Osteogenic Differentiation of *BMSCs*↑; Expression of *Alp, Bsp, an*d *OCN↑;* *B*one Regeneration: Complete filling of bone defects with new bone (21 d), BV/TV and *BMD*↑	2024[249]

GelMA is a representative hydrogel formulation that has been widely applied in various biomedical fields. It is a gelatin modified with double bonds, able to substitute artificial basement membranes due to its excellent biocompatibility, low antigenicity, and cellular response characteristics. More importantly, GelMA contains RGD (Arg-Gly-Asp) and MMP sequences, which play key roles in tissue repair and wound closure [250]. Additionally, GelMA can promote bone formation and angiogenesis [251]. As a photosensitive hydrogel, it undergoes cross-linking and solidification when irradiated with ultraviolet (UV) or visible light, providing numerous advantages, including good injectability and rapid gelation, making it highly suitable for bone tissue engineering [252]. Hydrogels typically exhibit relatively weak mechanical strength. To address this issue, bioceramic β-TCP has been widely utilized to optimize the mechanical properties and bioactivity of GelMA hydrogels [253]. Nie et al. incorporated β-TCP and sodium alginate into GelMA hydrogels as the base material for 3D printing. The introduced MXenes (a novel type of transition metal carbide/nitride/carbonitride) can effectively disrupt bacterial membranes through direct physical contact, thereby killing both G^+^ and G^−^ suspended bacteria and microbial biofilms, significantly enhancing antibacterial performance. This capability is further amplified under near-infrared (NIR) irradiation (808 nm). The authors filled 3D-printed GTAM scaffolds with rat *BMSCs*, which exhibited excellent biocompatibility and osteogenic capabilities in the presence of bacterial infection [241].

**Fig. 4:**
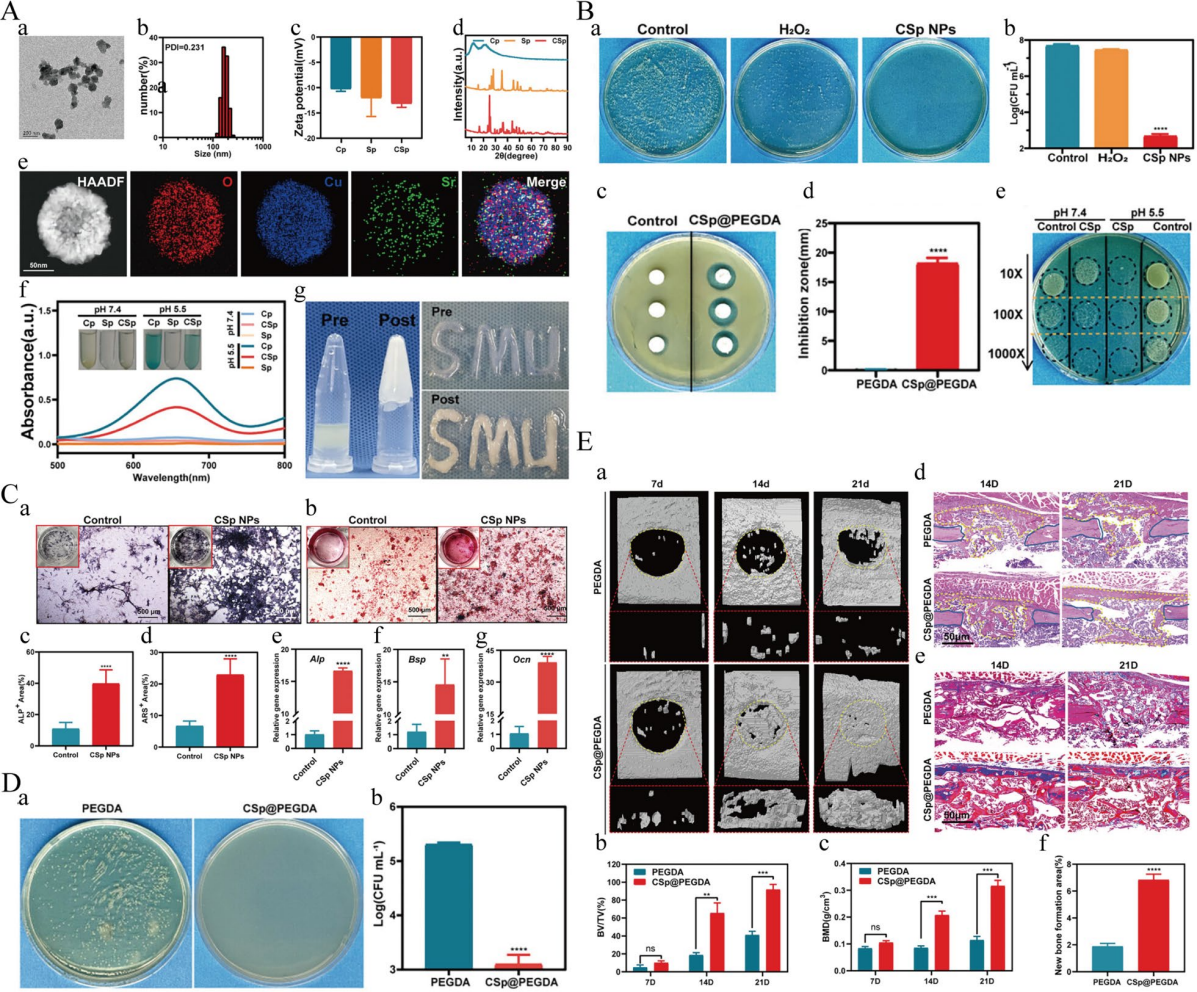
A schematic illustration highlights the synergistic antimicrobial and osteogenic effects of the CSp@PEGDA hydrogel. **A** Characterization of CSp@PEGDA. (a) TEM images, (b) Hydrodynamic diameter, (c) Zeta potential, (d) XRD patterns of CSp NPs. (e) HAADF image and element-mapping. (f) Absorption spectra of TMB oxidized by Cp, Sp and CSp NP at different pH. (g) Rapid gelation and plasticity of CSp@PEGDA under blue light irradiation (405 nm) for 30 s. **B** (a,b) Graphs and quantitative analysis of bacterial colony counts on TSA plates treated with CSp NPs. (c) Graph and (d) Quantitative analysis of inhibition zones of CSp@PEGDA composite on TSB agar plate. (e) Graph of bacterial colonies treated with CSp NPs at different pH conditions. **C** In vitro proliferative and osteogenic effect of CSp NPs on *BMSCs*. (a, c) *ALP* staining images of *BMSCs* co-cultured with CSp NPs for 7 days and their corresponding quantitative analysis. (b, d) ARS staining images of *BMSCs* co-cultured with CSp NPs for 21 days and their corresponding quantitative analysis. (e–g) Relative mRNA expression levels of osteogenesis-related genes *Alp (Da*y 7), *Bsp (Da*y 14), and *OCN (Da*y 21). **D** In vivo antimicrobial activity of CSp@PEGDA. Graphs(a) and quantitative analyses(b) of *S. aureus* in bone cavity rinses from CSp@PEGDA and PEGDA treated mice. **E** In vivo osteogenesis experiments. (a) Micro-CT images of mice femurs at days 7, 14, and 21. (b, c) Quantitative analyses of BV/TV and *BMD* at different time points. (d) H&E-stained sections of mice femurs at days 14 and 21, where yellow dashed lines indicate new bone formation, and blue solid lines mark the edges of bone defects. (e) Masson-stained sections of mice femurs at days 14 and 21. (f) Quantitative analysis of newly formed bone tissue at day 14. Copyright © 2024 Wiley‐VCH GmbH [249]

Cu^2^⁺ not only exhibits excellent antibacterial properties but also serves as a bioinorganic ion that promotes angiogenesis, primarily inducing vessel formation through the *VEGF* and HIF-α pathways [254]. Based on this, Xu et al. developed a GelMA hybrid hydrogel, namely GelMA/GeP@Cu, which enhances vascularization during the bone regeneration process. Its core component is GeP nanosheets modified with Cu^2^⁺, which not only support neurovascular regeneration but also possess antimicrobial properties. Experimental results indicate that this novel biocomposite hydrogel scaffold can continuously and slowly release Cu^2^⁺, inhibiting bacterial infection while promoting osteogenic cell differentiation and angiogenesis, thus facilitating bone tissue regeneration in infectious bone defect models [243]. A research team led by Guan et al. developed a composite hydrogel composed of copper-strontium peroxide nanoparticles (CSp NPs) and polyethylene glycol diacrylate (PEGDA) for the treatment of infectious bone defects. This innovative material leverages the pH-responsive release of hydroxyl radicals from Cu^2+^ within the hydrogel to achieve a 99.94% bacterial killing rate against *S. aureus*. Additionally, the hydrogel releases Sr^2+^ to enhance osteogenesis, resulting in complete bone defect repair in *IAOM* mice model within 21 days. This dual-functional strategy represents a significant advancement in non-antibiotic therapies for bone infections and regeneration, providing a promising clinical approach to combat drug resistance and promote bone healing (Fig. 4) [249].

In addition to the dual-functional study, Huang et al. constructed a hydrogel that integrates anticancer, antibacterial, MRI, and osteogenic functions [242]. They utilized the luminescence imaging advantage of Gd by incorporating Gd compounds and MoS₂ into GelMA. The MRI effect of Gd allows for monitoring the location and degradation of the hydrogel. MoS₂ endows the hydrogel with excellent photothermal capability, resulting in outstanding antibacterial and anticancer performance. As a rare earth element, Gd possesses an ionic radius similar to that of calcium ions and demonstrates good osteogenic ability in infectious bone defect models, presenting significant clinical translational value.

However, hydrogels have relatively weak mechanical strength, which may lead to damage and structural failure when used in bone tissue engineering due to daily activities. This can cause the loaded drugs to be released too quickly or to lose efficacy, thereby reducing treatment effectiveness [255]. Therefore, hydrogels with short-term self-healing capabilities improve this deficiency, extending their lifespan and enhancing safety [256].

Chen et al. combined the potent chemokine *SDF-1*, which can recruit bone marrow-derived osteoprogenitor cells, with exosomes secreted by *M2* macrophages and 4% HA, which exhibits strong antibacterial activity against *S. aureus*, *E. coli*, and methicillin-resistant *Staphylococcus aureus* (*MRSA*), to synthesize a self-healing adhesive hydrogel that is injectable, termed HA@SDF-1α/*M2*D-Exos. This hydrogel can restore itself in just 5 min after being divided into two parts, providing an effective solution to the problem of mechanical strength. The positively charged quaternary ammonium groups in the 4% HA effectively eliminated over 95% of *MRSA* and over 99% of both *S. aureus* and *E. coli*, while various osteogenic-related cells showed no significant cell death. In bone defect models, treatment with this hydrogel significantly enhanced angiogenesis and osteogenesis [257].

In addition to serving as the main component of composite materials, hydrogels can also play a significant role in collaboration. Three-dimensional (3D) printed metallic implants can be customized according to the clinical treatment needs of bone defects and the actual anatomy, aiming to restore the functionality of the original anatomical structure as much as possible [258]. For example, the surface micro-porous structure of porous titanium alloy (pTi) is more compatible with the host bone tissue, promoting angiogenesis and inducing inward bone growth. However, the porous structure may also become a breeding ground for bacteria, presenting significant vulnerabilities in the treatment of infectious bone defects [259]. Additionally, the ability of metal implants to induce cell proliferation and differentiation is relatively weak [260]. Therefore, Qiao modified the surface of pTi with an antibacterial hydrogel composed of sodium tetraborate (Na2B4O7), polyvinyl alcohol (PVA), AgNPs, and tetraethyl orthosilicate (TEOS). In vitro and in vivo studies that examined the inhibition of bacteria and the induction of *BMSCs* differentiation and mineralization indicated that this composite is an ideal candidate for promoting new bone formation in infected defects [261].

### Functionalized MOFs materials

In recent years, Metal Organic Frameworks (MOFs) with outstanding performance have shown great application potential in biological functional materials. MOFs are a class of porous inorganic–organic hybrid materials, synthesized through coordination between metal ions or clusters and organic ligands, also known as coordination polymers. They possess the rigidity of inorganic materials and the flexibility of organic materials [262–264]. MOFs exhibit high tunability, and their high specific surface area and adjustable pore structures allow for the encapsulation of a large number of functional molecules, facilitating in vivo transport and enabling their roles in delivery and catalysis [265]. Some MOFs materials demonstrate catalytic properties, such as peroxide catalysis, radiation sensitization, microwave heating, and photocatalysis [266, 267]. In addition, these materials can combine with other substances to form coatings or 3D structures, enabling a wider range of applications [268]. In the treatment of infectious bone defects, MOFs with good biocompatibility exhibit unique effects (Table 2).

**Table 2:** Strategies for the management of infectious bone defects employing MOFs

Categories	Components	Characteristics of MOFs	Assessments of infection	Assessments of bone healing	Year
Porphyrin- Zr -based (HN25 Sonosensitiz-er)	Alendronate	Morphology: diamond Zeta: 34.64 mV Surface Area: 1273.47 m^2^/g	Against *MRSA*: 98.97%; Animal models: Significant reduction in bacterial infection and abscess formation	CT and histological evaluations: Significant new bone formation and reduced bone defects; Osteogenic markers (*OPN*, *Runx2*, *OCN*) & *ALP* and ARS staining↑;	2024[269]
MOF-801 Zr -based	Ti、hydrofluoric acid (HF)	Coating thickness: Zr-MOF0, Zr-MOF1, and Zr-MOF2 were 5.38 μm, 9.63 μm, and 14.13 μm	The fluorine-doped Zr-MOF2 exhibited a bacterial inhibition rate of 79.43% and 66.28% against *S. aureus* and *E. coli*	Zr-MOF2 promoted *MC3T3-E1* proliferation and differentiation and induces macrophage transition to *M2*	2022[270]
UiO-66 Zr -based	Fosfomycin (FOS) CS-based scaffolds	Size:130 ± 28 nm	Antibacterial Activity: Complete bactericidal activity against *S. aureus*; Bacterial Attachment↓	Osteogenic Differentiation : Related genes (*RunX2*, *COL-I*, and *OCN*) and ECM mineralization in *MC3T3-E1* pre-osteoblasts↑; Gene Expression: COL-1*2 times, OCN*2.6–3.5 times↑; *ALP* Activity↑	2022[271]
Zn-base ZIF-8	Ag^+^ PEEK	Size: 300 nm Zeta: −19.4 mV	Colony Forming Units and Bacterial Growth Curve of SPZA indicated complete bactericidal effect	SPZA showed 85.0% cell viability against *L929* mouse fibroblast cells	2021[272]
Zn-base ZIF-8	CS	Rhombic Dodecahedron Size: 60 ± 20 nm Encapsulation Efficiency (*VAN*) : 99.3%	pH = 5.4(70% of *VAN* released with 8 h), pH = 7.4 (55%) inhibition zones:8–10 mm and a reduction index of ≥ 3	*MC3T3-E1* preosteoblasts showed high proliferation and osteogenic activities; *ALP* Activity and Calcium Deposition↑	2019[273]
Cu-base	SiO_2_-CaO AgNPs	Surface Area: 230 m^2^/g Pore Volume: 0.10 cm^3^/g	Antimicrobial Activity: inhibition zones between 8–10 mm and a reduction index of ≥ 3 MIC and MBC: 62.5 µg /mL to 125 µg/mL	Bioactivity: HAp formation after 7 days Cytotoxicity: No significant cytotoxic effects Cell Migration: Ability similar to control assays	2024[274]
Co-base ZIF-67	Osteogenic Growth Peptide (OGP), Titanium Dioxide Nanotubes (TNTs)	Rhombic Dodecahedron Size:189 ± 21 nm pH-sensitive property: rapid dissolution under acidic conditions	*E. coli* and *S. aureus* antibacterial ratios reached 94.9% and 94.5%, respectively; In vivo Antibacterial Efficacy(rat): fewer residual bacteria after 3 days of implantation	Osteointegration: a new bone formation area percentage of 42.6% and a bone-implant contact (BIC) ratio of 56.3% after 4 weeks; *M0 → M2, TNF-α*and *IL-1β*↓; *IL-10* and *IL-1* ↑	2021[275]
Mg-base	Calcium Sulfate, Calcium Citrate, Dicalcium Hydrogen Phosphate Anhydrous (DCPA)	Block structure 10–15 μm in size UV Absorption Peak: Characteristic peak at 259 nm	Antibacterial Activity: *S. aureus* survival rate ≤ 10% within 4 h Inhibition Zone: 17.75 mm and 20.59 mm (*S. aureus* and *E. coil*)	Mechanical Strength: increased cement strength from 27 to 32 MPa Osteogenic Differentiation: Promoted *mBMSC* proliferation and differentiation; Increased expression of *Runx2, BMP2, OCN, OPN,* and *COL-1* Inflammation Regulation: Reduced *IL-6* and increased *IL-10*	2023[276]

In response to the demand for various antibacterial strategies, MOFs through ion mediation, or based on physical methods, particularly showing good efficacy against antibiotic-resistant bacterial infections [277]. Among them, Zn-based MOF materials are excellent choices for treating infectious bone defects. Biocompatible ZIF-8, which has acid-responsive degradation properties, can degrade in response to acidic metabolites produced by bacteria. The released Zn^2^⁺ ions can penetrate the bacterial membrane through ion channels, thereby interfering with the bacteria [273]. Based on its favorable internal space, Han et al. incorporated *VAN* into ZIF-8 and deposited it onto 3D-printed bioglass scaffolds to bestow the composite material with bone repair functionality. This combination demonstrates a faster release rate of *VAN* and shows significant inhibitory effects on *S. aureus*. Furthermore, additional studies indicate that the scaffolds promote the expression of *MSCs* and osteogenic genes [278].

Research on MOFs materials using ion release strategies to combat infections and promote bone repair is quite abundant [270, 279]. Tao et al. coated ZIF-67 (Co-MOF) loaded with osteogenic growth peptides onto the surface of Ti to enhance its antibacterial properties and performance [275]. Due to the inert surface of PEEK limiting its clinical applications, Xiao et al. used a polydopamine (PDA) interlayer to bond Zn-Mg MOF-74 to PEEK, followed by loading Dex. This composite PEEK exhibits good hydrophilicity and stability, inducing an alkaline microenvironment on the surface through the release of Mg^2^⁺ and Zn^2^⁺, demonstrating strong antibacterial activity against *S. aureus* and *E. coli*. Additionally, the metal ions, in conjunction with Dex, synergistically promote the biological activity and osteogenic differentiation of rat bone marrow mesenchymal stem cells, while Mg^2^⁺ can also induce vascular differentiation of human umbilical vein endothelial cells. In rat subcutaneous infection models, chicken chorioallantoic membrane models, and rat femoral drilling models, this composite material shows good performance, providing new insights for the clinical translation of PEEK [279].

**Fig. 5:**
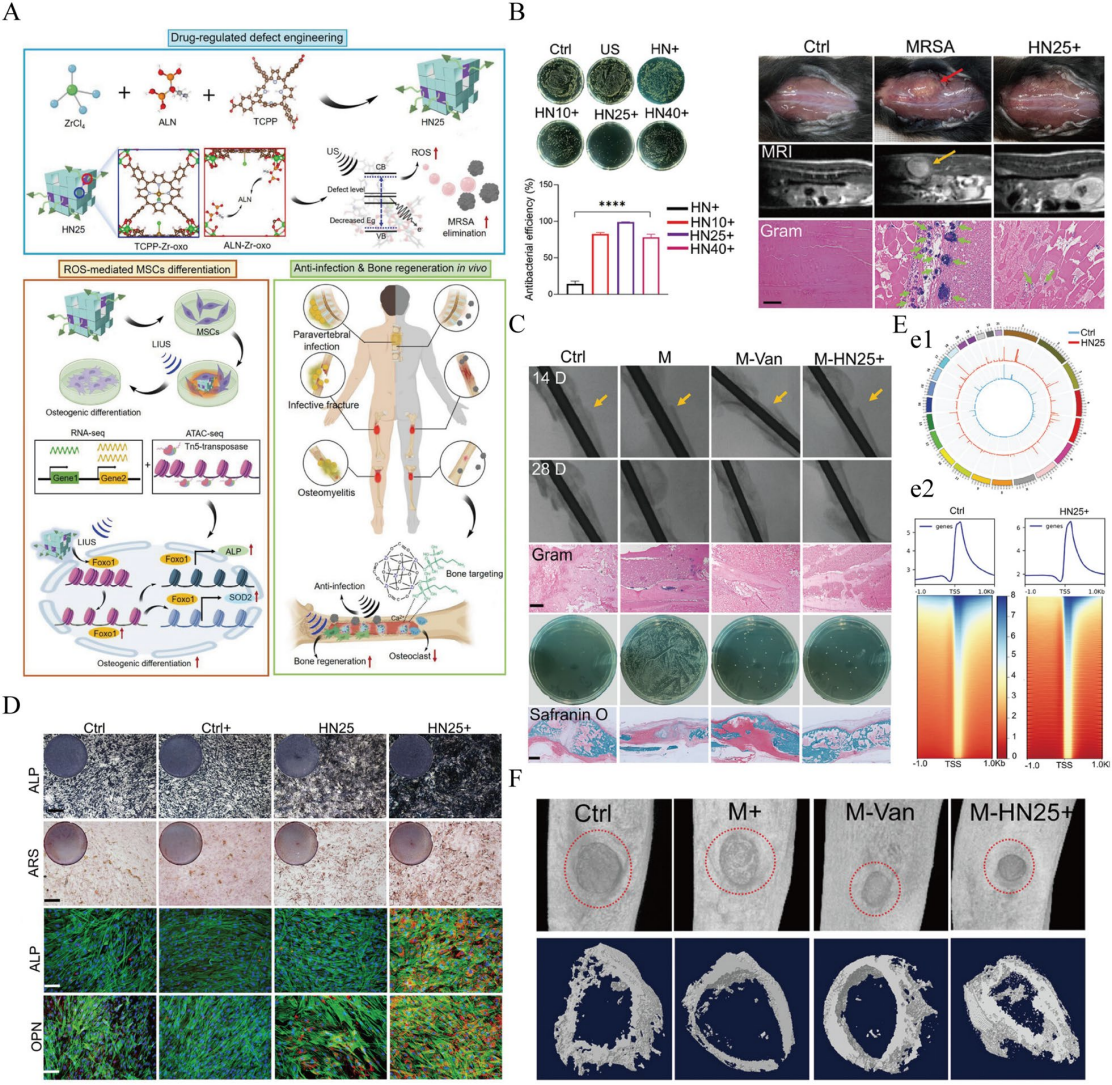
**A** This schematic illustrates the targeted repair of infectious bone tissue by the ALN-mediated defective MOF (HN25) through Sono-epigenetic modulation of chromatin accessibility, highlighting the dual role of HN25 in enhancing sonodynamic antibacterial activity and promoting bone regeneration under low-power ultrasound irradiation. **B** Antimicrobial efficiency of HN25 against *MRSA* under ultrasonic irradiation. **C** Micro-CT, Gram staining (Scale bar = 100 µm), spread plate of *MRSA* and Safranin O and fast green staining (Scale bar = 1 mm) of bone fracture sites after 14 and 28 d. **D** *OPN* (D1), *ALP* (D2) and ARS (D3) staining results demonstrate the ability of HN25 to promote osteogenic differentiation of *hMSCs*. **E** Osteogenesis mechanism. (e1) Genome-wide chromatin accessibility of *MSCs* after HN25 under US irradiation treatment and control. (e2) Heatmap of ATAC-seq signals enrichment at TSS regions in *MSCs* cells. **F** Illustration of anti-infection and bone regeneration of HN25. Copyright © 2023 Wiley–VCH GmbH [269]

In addition to the previously mentioned photosensitive MOFs that convert O₂ into *ROS*, some MOFs also possess photothermal effects, such as Prussian blue nanoparticles (PBNPs) [280, 281]. Han et al. proposed an innovative idea by mixing PBNPs with CS to create a novel hydrogel. Under the electrostatic adsorption of this hydrogel, bacteria are captured and killed through the photothermal effect stimulated by 808 nm NIR. However, there are certain limitations in the tissue penetration capability of light, which poses challenges for clinical translation [282]. Some sterilization materials using sonodynamic therapy based on MOFs have been reported, and their penetration is comparatively stronger than that of PTT. Ma et al. developed a novel composite material, HN25, which exhibits strong ultrasound-responsive antibacterial properties (Fig. 5). This material demonstrated significant antibacterial activity against *MRSA*, achieving an antibacterial rate of over 98.97% for *MRSA* within 4 h. Furthermore, HN25 has been shown to stimulate bone regeneration by promoting the osteogenic differentiation of mesenchymal stem cells, as evidenced by the increased expression of key bone-related genes and proteins such as *Runx2*, *OCN*, and *OPN*. Additionally, the composite exhibited biocompatibility with human dermal fibroblasts and the capacity to modulate inflammatory responses, positioning it as a promising candidate for bone repair applications without the need for surgical intervention or antibiotics [269].

In summary, the dual functions of antibacterial activity and osteogenesis in functionalized MOFs-based biomaterials are achieved through various properties of MOFs, including biodegradability, drug delivery, ion release, and their combined utilization. Moreover, the increase in antibacterial capacity aids in promoting osteogenesis, as inhibiting and eliminating infections in bone tissue contributes to repair [283]. Regardless of the antibacterial strategy employed, controlled characteristics must be achieved, such as sustained release of ions and drugs, as well as modulation of photothermal effects. Although many studies have demonstrated good antibacterial efficacy and osteogenic capability, there are still some issues regarding the controllability of antibacterial actions, particularly concerning the stability of the MOFs used in functionalized biomaterials [284, 285]. Therefore, further research and exploration of the biodegradability of MOFs are needed to better achieve the functionalization of biomaterials, endowing them with the dual functions of infection resistance and promotion of bone repair.

### Functional solid bone tissue substitutes

Infectious bone defects often lead to an expansion of the defect space during debridement, necessitating filling materials to withstand the stresses at both ends of the defect. Solid scaffolds typically possess characteristics such as shape stability, complex structures, and diverse functions, with the most important being mechanical properties similar to those of bone tissue. Therefore, 3D printing technology can create patient-specific biomimetic bone repair scaffolds through precise design. However, the implants generated by 3D printing often have relatively limited functions, requiring modifications to meet the demands of bacterial infection control and bone regeneration [286]. Some experimental design ideas have already been discussed previously, and the following will provide a more systematic description of this technology.

Modification of implants includes the application of physical and chemical methods to alter their surfaces. For example, physical modification significantly affects hydrophobicity, van der Waals forces, and electrostatic interactions, thereby influencing the adhesion of bacteria to the implant surface. Nanofibers, tubes, and needles can penetrate bacterial cell membranes and induce cell death, while also imparting responsive functionalities to the materials [287, 288]. For instance, Mo et al. used argon plasma to create inclined and vertical nanostructures on semi-crystalline PEEK, both of which can physically kill the bacteria that come into contact with them. The sharp edges of vertically arranged nanosheets can directly penetrate and damage bacterial membranes, while inclined nanosheets capture the adhered bacteria through high affinity, using strong shear forces to cause severe deformation or even rupture of the bacteria. Comparisons through bone regeneration experiments indicated that inclined nanosheets create favorable conditions for the attachment, proliferation, and osteogenic differentiation of bone cells, thereby promoting the integration of bone implants [289].

Chemical modification is typically achieved through coatings and chemical bonding, such as incorporating substances with antibacterial and anti-inflammatory properties, including antibiotics, peptides, and metal ions [290]. Yavari et al. first employed selective laser melting technology to fabricate a biomimetic topological 3D titanium scaffold, which was then covered with CS and gelatin containing cationic and anionic groups, followed by the coating of *BMP*−2 and *VAN* on the scaffold surface. In vitro drug release experiments demonstrated that this multilayer coating method allows for a sustained release of *BMP*−2 and *VAN* for approximately 3 weeks, with the adhered bacteria completely eliminated by day 7 [291]. Furthermore, some studies have loaded *MSCs* into scaffolds, with the most common and effective method being the use of 3D scaffolds to guide the proliferation, differentiation, and secretion of cells, thereby promoting various signaling pathways [292]. Wang et al. encapsulated peripheral blood-derived mesenchymal stem cells (*PBMSCs*) and endothelial progenitor cells (PEPCs) in a 75:25 ratio within 3D printed biphasic calcium phosphate (BCP) scaffolds, and coated the scaffold surface with a layer of highly active nano-hydroxyapatite (nHA), constructing a composite scaffold (nHA/BCP-PBEPC/PBMSC). This combination maximized the expression of osteogenic and angiogenic markers. Microfil angiography was performed on experimental model rabbits at 6 and 12 weeks, revealing the formation of abundant neovasculature around the scaffolds. Furthermore, histological analysis indicated a significant increase in new bone formation and mineralization, demonstrating excellent quality potential [293].

### Multifunctional bioceramics scaffolds

Previous descriptions have highlighted the excellent performance of bioceramics in promoting osteogenesis; however, almost all types lack antibacterial properties. Therefore, external antibacterial strategies must be employed to address infections related to bone implants. Currently, various composite bioceramic scaffolds have been developed and utilized for the treatment of infectious bone defects (Table 3).

**Table 3:** Composite Bioceramics scaffold design schemes for the treatment of infectious bone defects

Categories	Components	Strategy	Assessments of infection	Assessments of bone healing	Year
Mesoporous Bioactive Glass (MBG)	*DOX*; Bioink; PCL	*DOX*-Controlled *BMP*2 Expression;	*Vitro*: Significantly reducing bacterial adhesion and proliferation; *Vivo*: No bacterial fluorescence signals at 3 days	*Vitro*: *ALP* and Alizarin Red staining: Osteogenic differentiation of *BMSCs*↑; *Vivo*: X-ray, micro-CT, and histopathologic analysis: Significant ectopic bone formation; *BMP*2 Secretion↑	2020[294]
MSNs	Levofloxacin; HAp; Polyurethane (PU)	Controlled Release	Significant inhibition of *E. coli* and *S. aureus*; a visible zone of inhibition (*S. aureus*)	Osteogenic Differentiation: Expression of *ALP*, *OCN*, and *OPN* ↑; *MC3T3-E1* cells ↑; Cells were more in S and G2 phases (Cell Cycle Analysis)	2019[295]
HAp	PLGA; Linezolid	3D Printing	*Vitro*: Significant inhibition against *MRSA* (up to 28 days) *Vivo*: Viable bacteria in rabbit model↓	Micro-CT: Significant healing at the defect area, Bone trabeculae↑; H&E and Masson staining: Tissue destruction and Pus cell accumulation↓; Expression of osteogenic genes (*Runx2*, *OCN*, and *COL-1*) ↑	2024[296]
HAp; Calcium Sulfate (CaS)	Rifampicin (RFP)	Local Drug Delivery; Osteogenic Potential	Colony-forming units (CFUs) of *S. aureus*↓;	Micro-CT: BV/TV ↑; Histological Analysis: H&E and Masson staining indicated osteoconduction and bone regeneration ↑	2020[297]
β-TCP	Nanosized Silver (NSAg)	Controlled Silver Ion Release	Significant inhibition of *S. aureus* and *E. coli* growth;	Mechanical Strength: 7.74 ± 0.19 MPa; Histological Analysis: New bone formation rates per unit area were 18.6 ± 10.3%	2020[298]
β-TCP; S53P4 Bioactive Glass (BG)	Tea Tree Oil (TTO)	3D Printing; Coating Procedures	Significant antibacterial effects against *S. aureus*	Compressive strength of the scaffold was 2.4 ± 1.0 MPa; Viability of *MG-63* cells↑	2023[299]
β-TCP	Gelatin; CS; Gentamycin	Sustained Release	Inhibited the growth of *S. aureus* (in a dose-dependent manner)	The scaffold fused with bone tissues, and new tissues were formed in defect areas without any infection	2022[210]
α/β-Tricalcium Phosphate (α/β-TCP)	Tea Polyphenol-Magnesium (TP-Mg); Gelatin; Polyvinyl Alcohol (PVA)	Low-Temperature 3D Printing	*Vitro*: Inhibition rates of over 70% for *S. aureus* and nearly 60% for *E. coli*; *Vivo*: A significant reduction in bacterial colonies in a rat model of infectious bone defects; *M1* → *M2*	*Vitro*: Osteogenic differentiation of *BMSCs*↑; *Vivo*: Micro-CT showed BV/TV and *BMD*↑ (6 and 12 weeks)	2024[300]
Silicocarnotite (Ca_5_(PO_4_)_2_SiO_4_, CPS)	Germanium dioxide (GeO_2_)	Ultrasound-assisted aqueous precipitation method	Effectively inhibited the proliferation of *E. coli*) *S. aureus*; Colony Counting Assay: GeO2↑, Colonies↓; An inhibition ratio of *S. aureus* and *E. coli* were 72.1% and 76.4%, separately	Cell viability of *BMSCs*↑; Bone tissue regeneration and integration↑	2023[301]

**Fig. 6:**
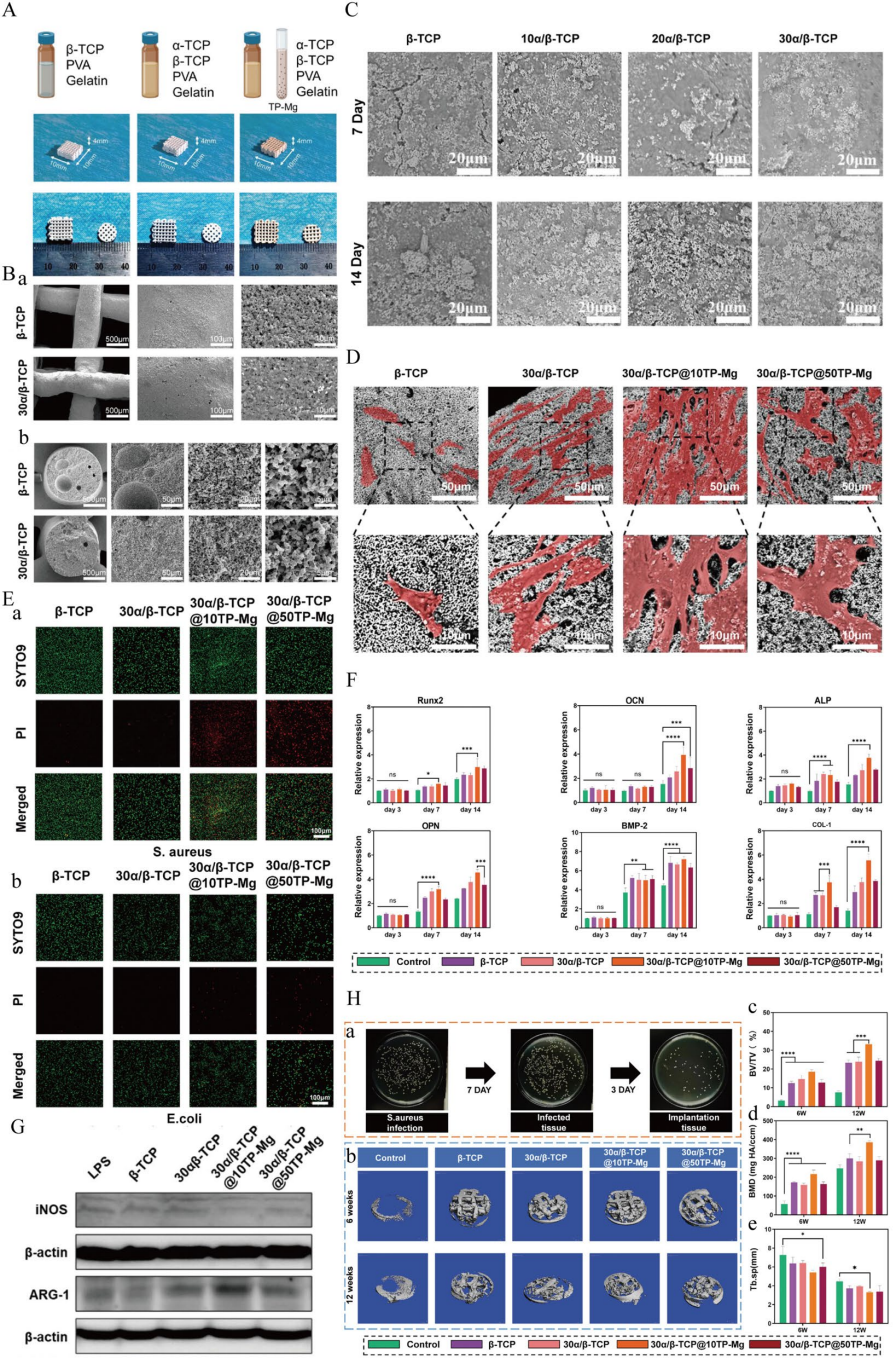
**A** Representative image of the scaffold. **B** SEM images of the scaffold showing (a) the top view and (b) the cross-sectional view. **C** Mineralization performance of the scaffold in simulated body fluid on days 7 and 14. **D** SEM image of *BMSCs* cultured with the scaffold for 7 days. **E** Fluorescence images showing live/dead bacteria of the scaffold against (a) *S. aureus* and (b) *E. coli*. **F** qRT-PCR analysis of the mRNA expression of osteogenesis-related genes *Runx2*, *OCN*, *ALP*, *OPN*, *BMP*−2, and *Col-1* on days 3, 7, and 14 of culture. **G** Western blot analysis of the expression of *M1* marker iNOS and *M2* marker ARG-1 in RAW264.7 macrophages cultured with the scaffold. **H** In vivo anti-infection and osteogenic capabilities: (a) Images of infected tissues at different time points; (b) Micro-CT images of rat calvaria after 6 and 12 weeks of treatment; (c) Quantitative analysis of BV/TV, (d) *BMD*, and (e) Tb. Sp. Copyright © 2024 The Author(s). Small published by Wiley‐VCH GmbH [300]

For instance, Wang et al. loaded doxycycline (*DOX*) into mesoporous bioglass (MBG) and then mixed it with molten PCL. Release experiments indicated that *DOX* rapidly released 150 μg on the first day, followed by a slow release, with concentrations reaching 400 μg and 600 μg on days 7 and 21, respectively. This composite scaffold significantly inhibited bacterial activity and enhanced its broad-spectrum antibacterial capability. Additionally, it also achieved notable improvements in promoting the differentiation of osteoblasts, which may be related to research showing that *DOX* at a dosage of 1000 ng/mL significantly stimulates *BMP*−2 expression [294]. Furthermore, antibiotics such as levofloxacin, gentamicin, chlorhexidine, berberine, and rifampicin have also been extensively studied in functionalized Bioceramics scaffolds, achieving favorable therapeutic effects [295, 297, 302–304]. Hu et al. developed a multifunctional biomimetic bone scaffold using 3D printing, which is made of α/β-TCP, Gelatin, PVA and loaded with TP-Mg nanoparticles. This scaffold exhibits significant antibacterial activity against *S. aureus* and promotes the polarization of macrophages from the pro-inflammatory *M1* phenotype to the anti-inflammatory *M2* phenotype. Additionally, the scaffold demonstrates excellent osteogenic effects through the synergistic action of Mg^2^⁺ and Ca^2^⁺. The study successfully developed a biomimetic bone scaffold that integrates anti-inflammatory, antibacterial, and osteogenic induction functions. This scaffold can regulate the early microenvironment and promote bone regeneration and healing, showing promising potential for the treatment of infectious bone defects (Fig. 6) [300].

In addition to the most commonly used antibiotics, other drugs, ion release, and physical effects (such as light, heat, and sound waves) have also become research hotspots [298, 305, 306]. The ions most commonly used for ion release include Ag⁺, Zn^2^⁺, Cu^2^⁺, La^3^⁺, and related oxidizers. These ions are incorporated into and coated onto bioceramics. The key point is that these excellent dopants are biocompatible with normal cells, while exhibiting lethal effects on drug-resistant bacteria, and they do not interfere with the effective osteogenesis of the scaffold [307–309]. Under the piezoelectric effect, ceramics can generate positive charges, thereby inducing antibacterial activity. Sugimoto et al. have developed a lead-free piezoelectric material (Ba, Ca) (Ti, Zr) O₃ (BCTZ50) for bone tissue engineering to enhance antibacterial performance. This material can generate a microelectric field, stimulating the surrounding liquid to decompose and produce *ROS*, thereby exerting a bactericidal effect [310].

### Functional two-dimensional (2D) biomaterials

2D nanomaterials possess ultra-thin structures and unique properties, providing new approaches for the treatment of infectious bone defects. Their thickness is only a few angstroms or nanometers, while their length and width can reach the micrometer level. This structure endows them with certain characteristics that 3D materials lack, such as a larger specific surface area, high thermal conductivity, and unique optical properties, including broad light absorption and efficient photothermal conversion [311]. Among these, materials like graphene and its derivatives, MXenes, BP, hexagonal boron nitride (hBN), and transition metal dichalcogenides (TMDs) have been extensively studied for treating bone diseases (Table 4).

**Table 4:** Research on the application of 2D materials in the treatment of infectious bone defects

Categories	Components	Strategy	Assessments of infection	Assessments of bone healing	Year
MXenes (Ti_3_C_2_)	Berberine (BBR); Biphasic Calcium Phosphate (BCP); Solidum Alginate (SA)	Phototherm-al Therapy (PTT);	*Vitro* Assay: Complete bactericidal activity against *S. aureus* after 96 h	Expression of osteogenic genes (*OCN*, *OPN*, *ALP*)↑; Superior BV/TV; Histological Analysis: H&E and Masson’s staining revealed more new bone formation and tissue regeneration in 1 and 4 months	2024[312]
MXenes (Ti_3_C_2_)	Vanadium Tetrasulfide (VS_4_): A semiconductor sonosensitizer (capable of generating *ROS*)	Ultrasound-Responsive Sonodynamic Therapy (SDT)	An antibacterial efficiency of 94.03% against *MRSA*; Inflammation↓	Expression of osteogenic genes (*OPN* and *ALP*)↑; Alizarin Red S (ARS) Staining: Richest Mineralized nodes; Bone Regeneration (*Vivo*): Significant new bone growth and mineralization;	2024[313]
MXenes (Ti_3_C_2_)	Peek + H_2_SO_4_ = SPEEK; PDA; Calcium Peroxide (CaO_2_): Provided hydrogen peroxide (H_2_O_2_) and Ca^2+^	PTT and Photodynamic therapy (PDT)	Against *MRSA*:100%; Against *S. aureus* and *E. coli*: Similar high antibacterial rates; Eradicated Biofilm	Osteogenic Differentiation: *ALP* and mineral ECM↑; Osseointegration strength↑; Micro-CT revealed new bone regeneration around implants; Increased collagen deposition and new bone formation	2024[314]
MXenes (Ti_3_ C_2_)	PMMA Polyethyleneimine (PEI) PDA	PTT	*Vitro*: 1. Antibacterial Efficiency: *MRSA* (94.03%), *E. coli* (96.25%) 2.Lactate dehydrogenase (LDH) cytotoxicity assay: Severe bacterial membrane damage *Vivo*: No observable bacteria in Gram staining	*Vitro*: Osteogenic genes (*ALP*, *Col1*, *BMP*2, *Runx2*) and mineralization↑; *Vivo*:1. CT showed more new bone formation after 4 and 8w 2.Histological evaluation (H&E and Masson): Less inflammation and more new bone formation	2023[315]
BP BP@Mg (Magnesium-modified black phosphorus)	GelMA	PDT PTT	*Vitro*: Antibacterial efficiencies against *E. coli* and *S. aureus* > 94% (NIR 5 min) *Vivo*: Antibacterial efficiencies of *S. aureus* > 96.22% (rat model) H&E and Giemsa staining: Inflammatory cells and bacteria↓ (1 week)	Micro-CT:BV/TV↑ (8 week) Masson’s trichrome staining: Substantial new bone formation with numerous osteoblasts around the bone tissue Immunofluorescence Staining: Nerve growth factor (NGF), calcitonin gene-related peptide (CGRP), and *OCN*↑	2023[316]
BP	Zinc sulfonate ligand (ZnL_2_) HAp	Sequential Photothermal Mediation: 1. < 50 °C: Antibacterial therapy 2.40–42 °C: Bone regeneration	*Vitro*:99.8 ± 0.2% and 99.7 ± 0.1% sterilization efficiency against *S. aureus* and *E. coli* *Vivo*: 98.53% ± 0.42% antibacterial efficiency against *S. aureus*	Osteogenic Gene Expression: *ALP*, *OCN*, *OPN*, *Runx2*↑ *ALP* Activity and ECM Mineralization↑ *Vivo*: BV/TV, *Tb.N* and Tb.Sp↑	2021[317]
BP pBP(protected by PDA)	Poly (aryl ether nitrile ketone) (PPENK) GelMA/ Dopamine Methacrylate (DMA)	PDT Antitumor therapy	*Vitro*: Antibacterial rate of 85% against *E. coli* and over 70% against *S. aureus* Antitumor Model: Tumor volume↓	Osteogenic Differentiation: Expression of *ALP*, *COL-1*, *Runx2*↑ Bone Regeneration: Micro-CT showed BV/TV↑ Biomechanical Testing: The bonding strength↑	2023[318]
Graphene	PEEK Hap Stearyltrimethylammonium Chloride (STAC) Cisplatin	Chemo-PTT Sequential Treatment	Bactericidal Rate: Near-total eradication (*MRSA* and *E. coli* under NIR irradiation)	*Vitro*: Expressions of *ALP*, *OPN*, *OCN*, and *Col1α1*↑ *Vivo*: Micro-CT Showed BV/TV and *Tb.N*↑ Histological Analysis: New bone formation and larger areas of new bone tissue	2021[319]
GO	Alginate Sericin nHAp	Immunomodu-lation	Macrophage Polarization: induced *M2* polarization; Inflammation↓ and osteoimmune microenvironment↑; *Vivo* Inflammation↓	*Vitro*: *ALP* activity and mineralization nodule formation↑; *Vivo*: BV/TV↑ and Fibrous Tissue Formation↓ Micro-CT: bone defect repair↑	2023[320]
GO	Alginate Antisense DNA Oligonucleotides (ASOs): Inhibited transcription of *S. aureus*	Photothermal Conversion; Targeted Antibacterial Action; Injectable and Degradable Hydrogel	*Vitro*: A zone of inhibition of 10 mm *Vivo*: Bacterial load and inflammation↓ (Mouse model)	Growth and proliferation of human umbilical cord-derived mesenchymal stem cells (*hUMSCs*)↑; Bone Regeneration: High antibiofilm performance and good biocompatibility	2022[321]
MoS_2_	Carbon Nanotubes (CNTs)	Microwave Thermal Therapy (MTT); Microwave Dynamic Therapy (MDT)	*Vitro*: Antibacterial rates of *S. aureus* and *E. coli* were 99.97%; Live/Dead Bacteria Staining: Significant number; SEM and TEM Analysis: Severe rupture and excitation of internal material	Wright and H&E Staining: Bacteria, lymphocytes, Multinucleated cells↓; Masson and Alizarin Red Staining: Osteoblasts and mature bone tissue↑; X-ray Analysis: No significant periosteum reaction or bone spots; Blood Routine and Biochemical Tests: Good anti-inflammatory ability and biocompatibility	2024[322]

**Fig. 7:**
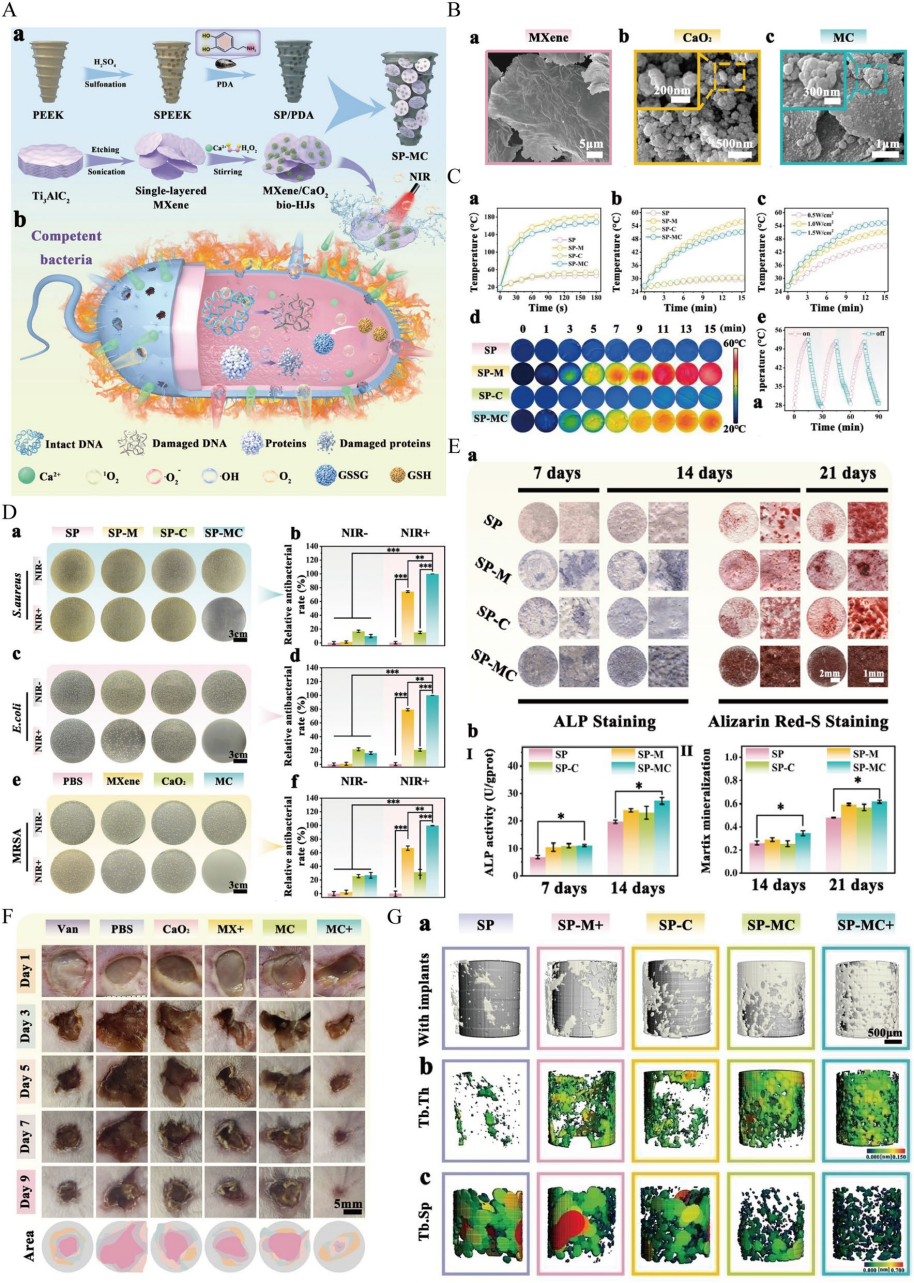
**A** (a) Schematic illustration of the synthesis of SP-MC. (b) Diagram of the anti-infection mechanism of SP-MC. **B** TEM images of (a) MXenes, (b) CaO, and (c) MC. **C** Photothermal and photodynamic performance of SP-MC. Under NIR laser irradiation, the temperature changes of different materials in (a) air and (b) PBS. (c) Temperature changes of SP-MC under different laser intensities. (d) Photothermal images of SP, SP-M, SP-C, and SP-MC. (e) Photothermal cycling curves of SP-MC. **D** In vitro antibacterial performance. Images and quantitative analysis of bacterial treatment with different materials: (a, b) *S. aureus*, (c, d) *E. coli*, and (e, f) *MRSA*. **E** Osteogenesis performance. (a) *ALP* staining and *ARS* staining results after the treatment with different implants. (b) The corresponding quantitative analysis of (I) *ALP* and (II) ARS. **F** Photos of infected wounds after different treatments. **G** In vivo osteogenesis performance assessed by Micro-CT. (a) Reconstructed images of newly formed bone on implants at 8 week, (b) Tb.Th, and (c) trabecular separation (Tb.Sp). Copyright © 2024 Wiley‐VCH GmbH [314]

MXenes exhibit good antibacterial activity, primarily due to their hydrophilic and anionic surfaces, which enhance interactions with bacterial cell membranes. The hydrogen bonds between their functional groups and lipopolysaccharide molecules hinder bacteria from absorbing nutrients, thereby inhibiting bacterial growth [323]. Yin et al. loaded tobramycin (TOB) into MXene-GelMA hydrogels, and the composite material showed significant destructive effects and promoted osteogenesis against *S. aureus*, maintaining this effect for over 24 h [324]. In one study, researchers functionalized MXenes with PDA to construct an alginate hydrogel that can harness NIR to control the photothermal heating behavior of the hydrogel. During the experiments, it was found that the composite material could regulate the conversion of *M1*/*M2* macrophages, thereby controlling inflammation and accelerating bone regeneration [325]. Huang et al. designed a nanostructure based on the MXene/CaO_2_ bio-heterojunction (MC bio-HJs), which possesses the functions of PTT and PDT. Under NIR irradiation, it can efficiently generate *ROS*, exhibiting nearly 100% antibacterial efficiency against *MRSA*, *S. aureus*, and *E. coli* (Fig. 7). Furthermore, the study revealed that MC bio-HJs achieves outstanding antibacterial effects by inhibiting bacterial energy metabolism and protein synthesis, thereby disrupting bacterial membrane function and metabolic processes. Both in vivo and in vitro experimental results further confirmed that MC bio-HJs significantly promotes the healing of infected skin wounds and bone repair, offering a novel therapeutic strategy to address the challenge of regenerative treatment for infectious bone defects [314].

BP is another promising 2D material for the repair of infectious bone defects, with related applications briefly mentioned in the previous sections. Recently, the fusion of BP with GelMA has also garnered widespread attention. Some studies have incorporated Mg^2^⁺ into black phosphorus nanosheets (BPNS) via electrostatic attraction, followed by merging with GelMA hydrogels. This approach validated the optimal concentration that significantly reduces the number of inflammatory cells and bacteria while promoting robust bone tissue regeneration [316]. Li et al. found that PDA significantly enhances the photothermal effect of BP hydrogels. Additionally, the controllability of drug release and encapsulation efficiency have also been optimized [318]. Wu et al. cleverly incorporated BPNS with zinc sulfonate ligands (ZnL₂) into HA scaffolds. The research results show that under mild photothermal conditions below 50 °C, it can effectively eliminate more than 99% of bacteria. The bactericidal mechanism may be due to the synergistic effect between ZnL₂ and positively charged BP, as well as the generation of *ROS*, leading to the destruction of intracellular biomolecules. Furthermore, the study also observed that the sustained release of Zn^2^⁺ combined with the presence of PO₄^3^⁻ jointly promotes the osteogenic process [317].

Other 2D nanosheets have also shown significant potential for clinical applications. Jin et al. utilized a heterojunction composite material composed of MoS₂ and CNTs. This MoS₂/CNTs composite demonstrated enhanced microwave absorption, which facilitated effective heat and *ROS* generation, enabling rapid bacterial clearance. Both in vitro and in vivo studies confirmed the composite's superior antibacterial activity, excellent biocompatibility, and ability to promote bone healing. These findings underscore its potential as a non-invasive treatment option for infectious bone defects [322].

Overall, a reasonable combination of various materials is the best approach to enhance the effectiveness of bone tissue engineering in treating infectious bone defects. This is also the direction that the majority of researchers are currently pursuing.

## Discussion

Bone infection is a complex process driven by interactions among various factors. In recent years, advanced diagnostic technologies have markedly improved early detection reliability. For example, α-defensin, an antibacterial peptide released by neutrophils upon activation, offers a biomarker for early infection identification [326]. Additionally, magnetic resonance imaging and nuclear medicine (such as technetium-99 m bone scans) are widely used to determine the anatomical location of osteomyelitis and the presence of associated abscesses, aiding in image-guided tissue biopsies. Additionally, novel identification technologies like matrix-assisted laser desorption ionization-time of flight mass spectrometry (MALDI-TOF MS) and genomic sequencing provide further insights into pathogen profiles and infection mechanisms [91].

Despite these advancements, treatment strategies for infectious bone defects continue to pose significant challenges (as discussed previously). The dual need for infection control and bone regeneration has driven the development of innovative biomaterial solutions. Bifunctional biomaterials have emerged as a promising alternative, offering enhanced clinical outcomes by supporting both bone regeneration and infection control at the defect site.

These materials typically involve surface modification and drug loading, enabling intelligent drug release through sustained release systems. They can release bioactive ions or molecules to kill bacteria, reduce inflammation, and promote osteoblast proliferation and differentiation, thereby accelerating bone tissue regeneration. As core scaffolds for bone repair, their design must meet mechanical performance requirements and possess good osteogenic activity and local anti-infection capabilities. In recent years, the development of composites has enhanced antibacterial effectiveness, biocompatibility, and mechanical performance, providing a stable and supportive environment for bone repair. Studies show that these materials can reduce postoperative infection rates, shorten bone healing times, and enhance bone strength.

Based on current research findings and clinical needs, an ideal bifunctional biomaterial should be able to rapidly release antibacterial components in the early stages of infection to inhibit the growth of pathogens. At the same time, it should continuously release factors that promote osteogenesis during the window of bone tissue regeneration to meet the treatment needs of different stages. Such precise temporal and spatial regulation can significantly enhance the effectiveness of infection control and bone healing. However, current research rarely addresses the selection of non-interfering drugs and the design of release modes, which may be a breakthrough point for future studies. Most existing research tends to focus on specific mechanisms, whereas infectious bone defects represent a complex pathological process involving multiple systemic contexts, suggesting that future investigations should consider the connections across various levels. Bifunctional biomaterials also present challenges in synthesis, stability, and large-scale production. Ensuring consistency and quality during production remains crucial for clinical application. Additionally, biosafety concerns, such as potential thrombosis or vascular injuries, warrant further investigation to ensure long-term viability.

## Conclusion

The advent of bifunctional biomaterials heralds a new era in treatment paradigms. These advanced materials are specifically designed to deliver targeted, phase-dependent antibacterial and osteogenic effects, demonstrating significant potential to markedly improve clinical outcomes for patients suffering from bone infections. We believe that with the continuous development of science and technology, the treatment of bone infections will be more efficient and robust, and these materials will offer more mechanically durable, functionally effective, and personalized solutions.

## Data Availability

No datasets were generated or analysed during the current study.
